# Evolution of Chaperonin Gene Duplication in Stigonematalean Cyanobacteria (Subsection V)

**DOI:** 10.1093/gbe/evw287

**Published:** 2017-01-12

**Authors:** Julia Weissenbach, Judith Ilhan, David Bogumil, Nils Hülter, Karina Stucken, Tal Dagan

**Affiliations:** 1 Institute of General Microbiology, Christian-Albrechts University of Kiel, Am Botanischen Garten 11, Kiel, Germany

**Keywords:** *groEL*/*groES*, Hsp60/Hsp10, heterocyst

## Abstract

Chaperonins promote protein folding and are known to play a role in the maintenance of cellular stability under stress conditions. The group I bacterial chaperonin complex comprises GroEL, that forms a barrel-like oligomer, and GroES that forms the lid. In most eubacteria the GroES/GroEL chaperonin is encoded by a single-copy bicistronic operon, whereas in cyanobacteria up to three *groES/groEL* paralogs have been documented. Here we study the evolution and functional diversification of chaperonin paralogs in the heterocystous, multi-seriate filament forming cyanobacterium *Chlorogloeopsis fritschii* PCC 6912. The genome of *C. fritschii* encodes two *groES/groEL* operons (*groESL1*, *groESL1.2*) and a monocistronic *groEL* gene (*groEL2*). A phylogenetic reconstruction reveals that the *groEL2* duplication is as ancient as cyanobacteria, whereas the *groESL1.2* duplication occurred at the ancestor of heterocystous cyanobacteria. A comparison of the *groEL* paralogs transcription levels under different growth conditions shows that they have adapted distinct transcriptional regulation. Our results reveal that *groEL1* and *groEL1*.2 are upregulated during diazotrophic conditions and the localization of their promoter activity points towards a role in heterocyst differentiation. Furthermore, protein–protein interaction assays suggest that paralogs encoded in the two operons assemble into hybrid complexes. The monocistronic encoded GroEL2 is not forming oligomers nor does it interact with the co-chaperonins. Interaction between GroES1.2 and GroEL1.2 could not be documented, suggesting that the *groESL1.2* operon does not encode a functional chaperonin complex. Functional complementation experiments in *Escherichia coli* show that only GroES1/GroEL1 and GroES1/GroEL1.2 can substitute the native operon. In summary, the evolutionary consequences of chaperonin duplication in cyanobacteria include the retention of *groESL1* as a housekeeping gene, subfunctionalization of *groESL1.2* and neofunctionalization of the monocistronic *groEL2 paralog.*

## Introduction

How new genes emerge and gain functionality is a fundamental question in biology. While the emergence of *de novo* protein-coding genes from noncoding DNA is extremely rare during evolution, new function often evolves from “molecular tinkering” of preexisting genes or parts of genes that are transformed and give rise to new function (Jacob 1977; Moyers and Zhang 2016). Gene duplication is considered as a major mechanism for the evolution of novel protein-coding genes and the development of phenotypic innovation (Ohno 1970; Lynch 2007). The amplification of gene copies is frequent during microbial evolution and the gene copy number can be highly transient within microbial populations (Romero and Palacios 1997). A temporary gene amplification may result in a dose effect where the protein encoded by the gene is produced in excess (e.g., Fuentes-Hernandez et al. 2015). The long-term retention of duplicated genes can involve, in addition to dose effect, subfunctionalization of the paralogous gene that retains a subset of the original function, or neofunctionalization of the gene duplicate into a novel function (Lynch 2007). Yet, paralogous gene copies are rarely retained during microbial evolution and most genes (89 ± 8%) in prokaryotic genomes are found in a single copy (Bratlie et al. 2010). In addition, the evolution of transcriptional regulation in bacteria may be rapid, leading to high plasticity of the regulatory elements that control the gene transcription level (Oren et al. 2014). Thus, the rapid adaptation of a new protein expression regime can often proceed through a modification of a regulatory region rather than gene amplification (e.g. Amoros-Moya et al. 2010). Hence, paralogous genes encoded in bacterial genomes constitute an exception from the common fate of duplicated genes in the prokaryotic domain.

Here, we study the evolution of chaperonin gene duplication in cyanobacteria. The chaperonin GroEL and its co-chaperone GroES function in unison to promote the folding of client proteins in an ATP-dependent manner (Horwich et al. 1993), while GroEL is also able mediate protein unfolding independently of ATP (Priya et al. 2013). Molecular chaperones are known to mediate stress response in many organisms where their expression is regulated according to various environmental cues including heat, UV radiation, salinity, and light stress (e.g. Webb et al. 1990; Glatz et al. 1997; Yamazawa et al. 1999; Chaurasia and Apte 2009). Moreover, accumulating evidence suggests that the function of chaperones has important consequences for robustness (Queitsch et al. 2002) and adaptation to high mutational load (Sabater-Muñoz et al. 2015). GroESL-mediated folding has been shown to accelerate protein evolution *in vitro* (Tokuriki and Tawfik 2009). This phenomenon is evident in genomic comparisons of eubacterial organisms as obligatory GroEL substrates were found to evolve faster than casual GroEL interactors (Bogumil and Dagan 2010; Warnecke and Hurst 2010; Williams and Fares 2010).

Chaperonins are encoded in most eubacteria (∼70%) by a single bicistronic *groESL* operon, while several taxa encode multiple paralogs, including Burkholderiales, Rhizobiales, Mycobacteria, and Cyanobacteria (Lund 2009). Cyanobacteria invented photosynthesis (Mulkidjanian et al. 2006) and they furthermore represent a rare example of genuine cell differentiation within prokaryotes. Cyanobacteria are classified into a monophyletic phylum that includes genera presenting a wide range of phenotypic diversity. Based on their cellular and colony morphology they have been divided into five subsections (Rippka et al. 1979). Under nitrogen-starvation (i.e. diazotrophic conditions), species of the Nostocales (Subsection IV) and Stigonematales (Subsection V) orders differentiate heterocyst cells that supply an oxygen-depleted environment for nitrogen fixation (Flores and Herrero 2010). All cyanobacterial genomes tested so far encode at least one *groESL* operon and a monocistronic *groEL* gene (Lund 2009). An exception is *Gloeobacter violaceus*, which encodes two *groESL* operons but no monocistronic *groEL* (supplementary table S1, Supplementary Material online). The recent genome sequencing of stigonematalean cyanobacteria revealed that species in that order encode a second copy of the *groESL* operon (Dagan et al. 2013). These species are characterized by multi-seriate or true-branching filament formation, the ability to fix nitrogen in heterocysts and differentiate morphologically distinct cell types (Rippka et al. 1979).

The functional role of cyanobacterial *groEL* paralogs has been studied so far mostly in the unicellular cyanobacterium *Synechocystis* sp. PCC 6803 and the filamentous, heterocystous cyanobacterium *Anabaena* sp. L31. In both species, the two *groEL* paralogs have been shown to be upregulated during heat stress (Glatz et al. 1997). Expression of the monocistronic *groEL* in *Synechocystis* is repressed during heat stress when photosynthesis is inhibited (Glatz et al. 1997). Furthermore, complementation experiments in *E. coli* showed that the monocistronic *groEL* of *Synechocystis* sp. and *Synechococcus vulcanus* cannot complement the native chaperonin (Kovács et al. 2001; Furuki et al. 1996; Tanaka et al. 1997). In *Anabaena* sp. L31, expression of the monocistronic *groEL* is repressed when heat stress is applied in diazothrophic conditions (Rajaram and Apte 2008). These studies, as well as studies in *Thermosynechococcus elongatus* (Sato et al. 2008) and *Synechococcus elongatus* PCC 7942 (Huq et al. 2010), thus suggest that the monocistronic *groEL* paralog has undergone a subfunctionalization while the bicistronic *groEL* paralog maintains a housekeeping function. Here, we study the fate of chaperonin gene duplication in stigonematalean cyanobacteria whose genome includes an additional *groESL* operon. We reconstruct the evolutionary history of chaperonin gene duplications in that order using phylogenetics. Moreover, we analyze functional divergence of the three chaperonin paralogs in Stigonematales with the cyanobacterium *Chlorogloeopsis fritschii* PCC 6912 as a representative. By comparing the transcription level and localization under different growth conditions, we show that the *groEL* paralogs have acquired a distinct transcriptional regulation. Furthermore, we examine the potential of GroES*/*GroEL paralogs to assemble into a chaperonin complex using protein–protein interaction studies and test for functional diversification of the paralogs by complementation of a *groESL* depleted *E. coli* strain. Our study suggests that the retention of *groES/groESL* duplicates in Stigonematales is accompanied by sub- and neo-functionalization of the paralogs.

## Results

### Evolution of *groEL* and *groES* Paralogs in Cyanobacteria

To study the evolution of *groES*/*groEL* paralogs in cyanobacteria we searched for all homologs of the chaperonin subunits in 141 sequenced genomes. Most of the cyanobacteria in our sample (115; 81%) encode a single *groESL* operon and an additional monocistronic *groEL* (supplementary table S1, Supplementary Material online). Cyanobacteria species that encode an additional *groESL* operon are mostly of the filamentous types included in Subsections III, IV, and V. Exceptions among the unicellular cyanobacteria (Subsection I) are *Synechococcus* sp. PCC 7335 and *Synechococcus* sp. PCC 7336 that encode two *groESL* operons. Within Subsection III, all three *Pseudoanabaena* strains encode two *groESL* operons, while *Leptolyngbya* sp. PCC 7375 encodes two *groESL* operons and two additional monocistronic *groEL* genes. Many heterocystous cyanobacteria that form linear or branching filaments (Subsections IV and V) encode an additional *groESL* operon (supplementary table S1, Supplementary Material online).

**Fig. 1.—:**
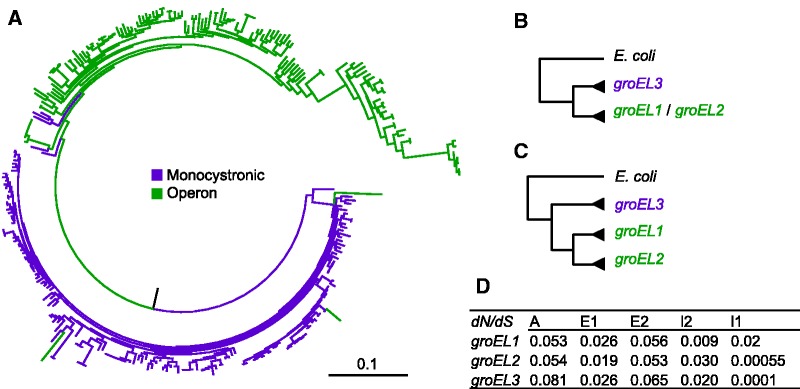
Phylogenetic reconstruction of *groEL* paralogs. (*A*) A ML phylogeny of *groEL* DNA sequences in 141 cyanobacteria. (*B*) Constrained topology used to test for an ancient duplication of the monocistronic and operon-encoded *groEL*. (*C*) Constrained topology used to test for duplication of *groESL1* and *groESL1.2*. (*D*) The relative rate of nonsynonymous and synonymous substitutions (dN/dS) in Stigonemalaes *groEL* domains. A: Apical (amino acids 160-375); E1: 5′ Equatorial (amino acids: 1-133); E2: 3′ Equatorial (amino acids 411-546); I1: 5′ Intermediate (amino acids: 134-189); E2: 3′ Intermediate (amino acids 376-411).

A maximum-likelihood phylogenetic tree reconstructed from all cyanobacterial homologous *groEL* DNA sequences reveals two main clades that largely correspond to the monocistronic and operon encoded genes (fig. 1*A*). Furthermore, most of *groEL* duplicates encoded in genomes that encode an additional operon (e.g. Subsection V) form a sub-clade that is nested within the operon encoded *groEL* clade. In the following, we term the genes encoded in the ubiquitous *groESL* operon by *groES1* and *groEL1*, while the additional operon components are termed *groES1.2* and *groEL1.2*. The monocistronic *groEL* is termed *groEL2*. To further study the evolution of *groES*/*groEL* paralogs we reconstructed phylogenies that include all strains encoding more than two *groEL* paralogs and classified the genes into the three named paralogs (supplementary table S1, Supplementary Material online). The tree topology suggests that the split between the operon encoded and monocistronic *groEL2* is ancient (fig. 1*B*). To test this hypothesis we reconstructed a constrained phylogeny where the split between the monocistronic and operon clades is fixed (fig 1*B*) and compared the likelihood of the resulting topology with the maximum likelihood (ML) phylogeny. This comparison revealed that the constrained phylogeny is not significantly different from the ML phylogeny (*P* = 0.35, using Shimodaira–Hasegawa (SH) test; Shimodaira 2002). Consequently, we conclude that the duplication of the ubiquitous operon *groESL1* and the monocistronic *groEL2* is ancient and most probably occurred at the base of the cyanobacterial tree.

We further tested for monophyly of the operon-encoded *groEL* duplicates by adding a constrained split between *groEL1* and the remaining *groEL* duplicates (fig. 1*C*). The likelihood of that topology is significantly lower than the ML phylogeny (*P* = 1 × 10^−^^61^, using the SH test). We note, however, that this topology includes also exceptional *groEL* paralogs that could not be classified into the three orthologous groups such as additional monocistronic *groEL* paralogs in *Leptolyngbya* sp. PCC 7375 and *N. punctiforme* PCC 73102. Testing for a constrained monophyly of *groES1* and *groES2* revealed that the constrained topology is not significantly different from the ML topology (*P* = 0.15, using the SH test). To further characterize the evolutionary dynamics of the *groES*/*groEL* paralogs in stigonematalean cyanobacteria we constructed a phylogenetic tree that includes all paralogs encoded in the Nostocales (Subsection IV) and Stigonematales (Subsection V) genomes in our sample (supplementary fig. S1, Supplementary Material online). The Nostocales–Stigonematales clade includes all heterocyst forming cyanobacteria and is considered monophyletic (Flores and Herrero 2010; Dagan et al. 2013). The *groES* phylogeny reveals a clear monophyly of *groES1* and *groES1.2* that is highly supported by bootstrap (supplementary fig. S1A, Supplementary Material online). Testing for a constrained topology where *groEL2* is monophyletic yielded a topology that is not significantly different than the ML phylogeny (*P* = 0.88, using the SH test). This result confirms that the duplication of *groEL1* and *groEL2* is ancient. A further test of the split between *groEL1* and additional *groEL* duplicates reveals a topology that is not significantly different than the ML phylogeny (*P* = 0.15, using SH test). This indicates that the duplication of *groESL1* operon occurred before the divergence of Stigonematales. Thus, the heterogeneous distribution of *groESL1.2* across the Nostocales and Stigonematales is most probably due to a single duplication event and differential loss of *groESL1.2* in the species that lack that operon rather than lateral gene transfer.

### Transcriptional Regulation of *groEL* and *groES* Paralogs in *C. fritschii* PCC 6912

Transcriptional regulation of the *groESL* operon and the monocistronic *groEL* in cyanobacteria is known to depend on the presence of CIRCE and K-box elements at the 5′-UTR (Kojima and Nakamoto 2007; Sato et al. 2008). Comparative genomics of the *cis*-regulatory elements upstream of the *groES/groEL* paralogs in Stigonematales reveals conserved CIRCE and K-box elements upstream of the *groESL1* operon (supplementary table S2, Supplementary Material online). The CIRCE element upstream of *groESL1.2* is slightly diverged and is absent in *Fischerella* sp. PCC 73103 and *Fischerella thermalis* PCC 7521. The *groEL2* is preceded in all genomes by a conserved CIRCE element but lacking a K-box element (supplementary table S2, Supplementary Material online). To study differences in the transcriptional regulation of the *groEL* paralogs, we quantified their transcription level in *C. fritschii* PCC 6912 using qRT-PCR. In addition, we visualized the promoter activity of the three paralogs in real time using a green fluorescence protein (GFP) marker.

**Fig. 2.—:**
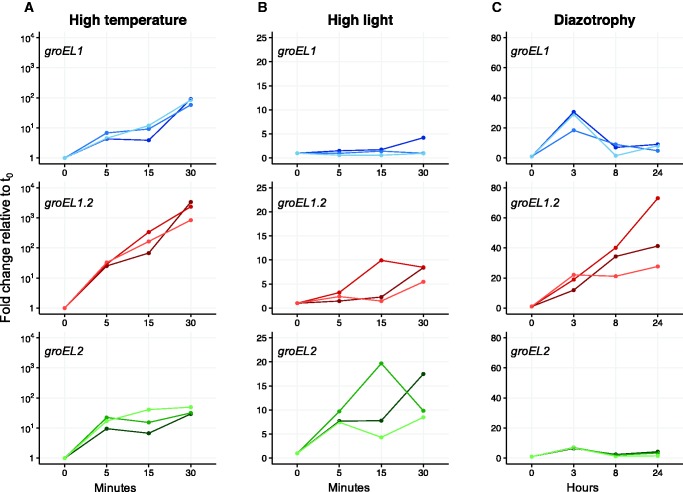
Relative transcript abundance of the *groEL* paralogous genes in *C. fritschii* PCC 6912 under different growth conditions. Three independent biological replicates are plotted. (*A*) High temperature (50 °C). (*B*) High light (70 µE m^−2^ s^−1^). (*C*) Diazotrophy (nitrogen deprivation). Transcript abundance is depicted as fold change relative to expression levels under standard growth condition (37 °C, 24 µE m^−2^ s^−1^).

The absolute transcript levels of the three paralogous genes are significantly different under standard growth conditions (*P *< 0.05, using paired *t*-test with Bonferroni correction). The *groEL1* transcript is the most abundant and *groEL2* transcript abundance exceeds that of *groEL1.2* (supplementary fig. S2, Supplementary Material online). We then compared the paralogs transcription level under high temperature by incubating *C. fritschii* at 50 °C. After 5 min of heat exposure (*t*_5m_) the transcription level of *groEL1* increased 5-fold on average, while the transcription level of *groEL1.2* and *groEL2* increased more than 15-fold, on average, in comparison to their transcription level under standard growth conditions (fig. 2*A*). After 15 min (*t*_15m_), the transcription level of *groEL1* increased 8-fold and that of *groEL1.2* increased 189-fold in comparison to the onset of the experiment (*t*_0_). The transcription level of *groEL2* remained similar to *t*_5m_. The transcription level of all three paralogs increased further after 30 min of incubation at 50 °C, where the highest increase of about 2,000-fold was observed for *groEL1.*2. The transcription level of *groEL2* increased 37-fold, whereas *groEL1* transcription increased 78-fold in comparison to *t*_0_ (fig. 2*A*).

**Fig. 3.—:**
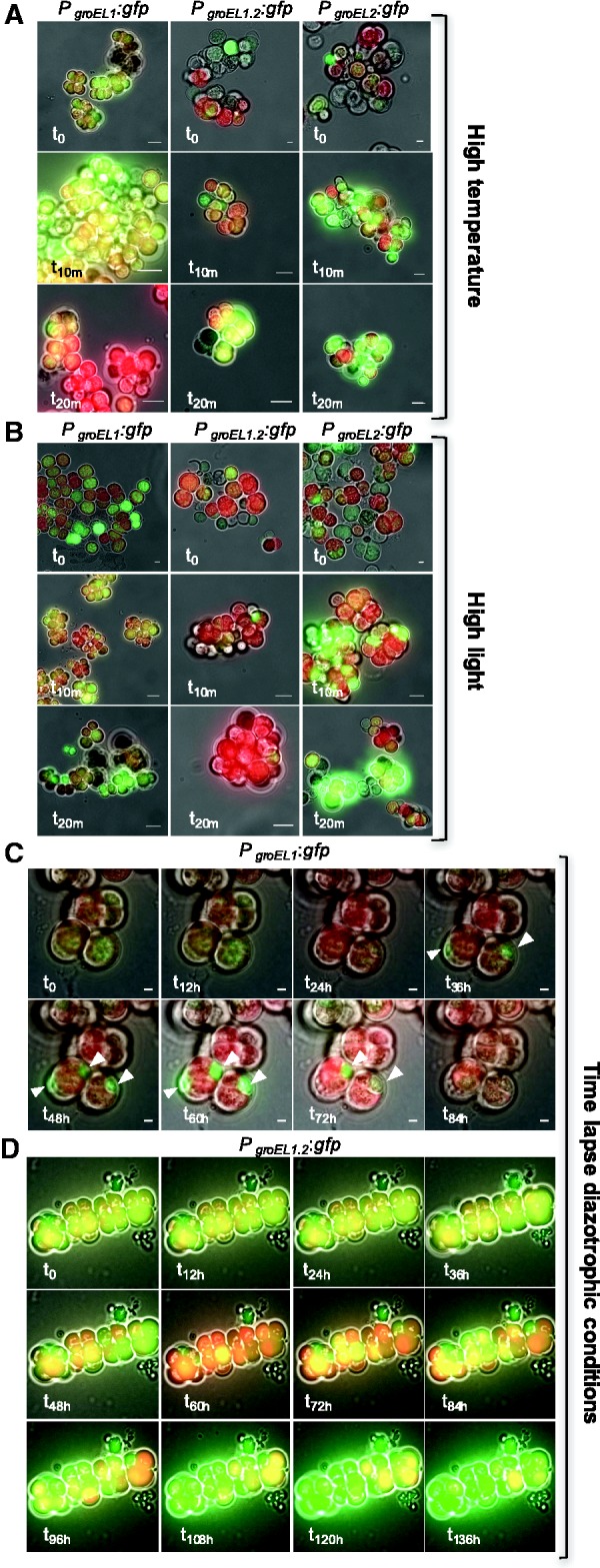
Expression of GFP under control of the different paralogous *groEL* promoters in *C. fritschii* PCC 6912. Micrographs of cyanobacterial cells expressing GFP under the transcriptional regulation of the three *groEL* promoters. For each fusion protein merged pictures of bright-field, GFP- (green), and chlorophyll fluorescence (red) are shown at different time points under various growth conditions (see fig. S4 for separate fluorescence signals). The time is indicated at the bottom left corner of each frame. Scale bars represent 10 µm. The white arrows indicate GFP accumulation in specific cells. (*A*) High temperature. (*B*) High light. (*C* and *D*) time-lapse of P_*groESL1*_- (*C*), and P_*groESL1.2*_- (*D*) driven GFP expression during diazotrophic conditions. Due to the different basal expression of both paralogs, the fluorescent intensity in (*C*) and (*D*) was measured with different light intensities and exposure times.

To further validate changes in the transcriptional regulation of the three paralogs under varying growth conditions, we created transcriptional fusions of each *groESL*/*groEL* promoter with *gfp-mut3.1* (*PgroESL1*:*gfp*, *PgroESL1.2:gfp*, *and PgroEL2:gfp*). The three transcriptional fusion constructs were introduced independently into *C. fritschii*. The GFP fluorescence was observed in all transformed strains grown under standard conditions (*t*_0_), indicating that all promoters are active and GFP-fusions are functional. During high temperature stress conditions *groESL1* promoter activity increased after 10 min (*t*_10m_), but decreased after 20 min (*t*_20m_) of incubation (fig. 3*A*). Elevated promoter activity of *groESL1.2* and *groEL2* could be clearly observed after 20 min (fig. 3*A*, supplementary fig. S4*A*, Supplementary Material online). Thus, promoter activity of the three paralogs as documented by GFP-fusion constructs supports the transcription level changes recorded using qRT-PCR.

To compare the transcriptional regulation of the paralogs under high light conditions, we incubated *C. fritschii* PCC 6912 under 3-fold higher light intensity compared with standard conditions (fig. 2*B*). The transcription level of *groEL1* did not change during the course of the experiment. The *groEL1.2* transcript level slightly increased after 30 min of incubation. A continuous increase of *groEL2* transcript abundance under high light conditions was observed, up to a maximum of a 12-fold change in comparison to *t*_0_ (fig. 2*B* and supplementary table S4, Supplementary Material online). The promoter activity of all paralogs, as documented by fluorescence intensity (fig. 3*B*; supplementary fig. S4*B*, Supplementary Material online), validates the observed changes in transcription level under high light conditions.

Heterocyst differentiation is a common trait in cyanobacteria with multiple *groESL* copies. Consequently, we compared the paralogs transcriptional regulation under diazotrophic conditions that induce heterocyst differentiation. Here we used the transcription level of glutamine synthetase (*glnA*) as a positive indicator for nitrogen deprivation (Tumer et al. 1983). After 3 h incubation under diazotrophic conditions the expression of *glnA* increased 10-fold, followed by a decrease observed after 8 h (supplementary fig. S31, Supplementary Material online). This was accompanied by an average increase of 26-fold in *groEL1* transcript abundance after 3 h followed by a decrease after 8 h (fig. 2*C*; supplementary table S4, Supplementary Material online). The transcription level changes of *groEL2* were similar to that of *glnA*, but with a maximum of 7-fold change after 3 h. The transcription level of *groEL1.2* increased 18-fold after 3 h and continued to increase with a maximum of 47-fold change after 24 h in comparison to t_0_. The transcription dynamics of *groEL1* suggest that it plays a role during the early stages of diazotrophy, while *groEL1.2* seems to play a role in later stages of the adaptation to diazotrophic conditions. Consequently, we tested the promoter activity of *groESL1* and *groESL1.2* under diazotrophic conditions by time-lapse fluorescence microscopy (fig. 3*C* and *D*). Because, *C. fritschii* grows slower on solid media in comparison to liquid media, we followed the promoter activity for a longer duration than the qRT-PCR experiment. During the first 24 h under diazotrophic conditions no specific promoter activity of *groESL1* expression was observed. The fluorescence pattern after 36 h suggests that increased expression of GFP from *groESL1* promoter leads to accumulation of GFP in specific cells that seem to differentiate into heterocysts in later stages (fig. 3*C*). After 72 h, the *groESL1* promoter appeared to be no longer active. The promoter of the second *groESL* operon (*groESL1.2*) showed an increased activity after 96 h followed by further elevation in all cells during the time of incubation in diazotrophic conditions (fig. 3*D*). These results correspond to the transcription dynamics of *groEL1* and *groEL1.2* as documented using qRT-PCR, where *groEL1* is upregulated in the early stage and *groEL1.2* is upregulated in the late response to diazotrophic conditions. Our results further indicate that *groESL1* promoter activity during diazotrophy is restricted to specific cells whereas *groESL1.2* promoter activity did not show any cell-specific localization. Overall, the differences we observed in the transcriptional regulation of the three paralogs suggest that they have undergone a subfunctionalization.

### Chaperonin Assembly

**Fig. 4.—:**
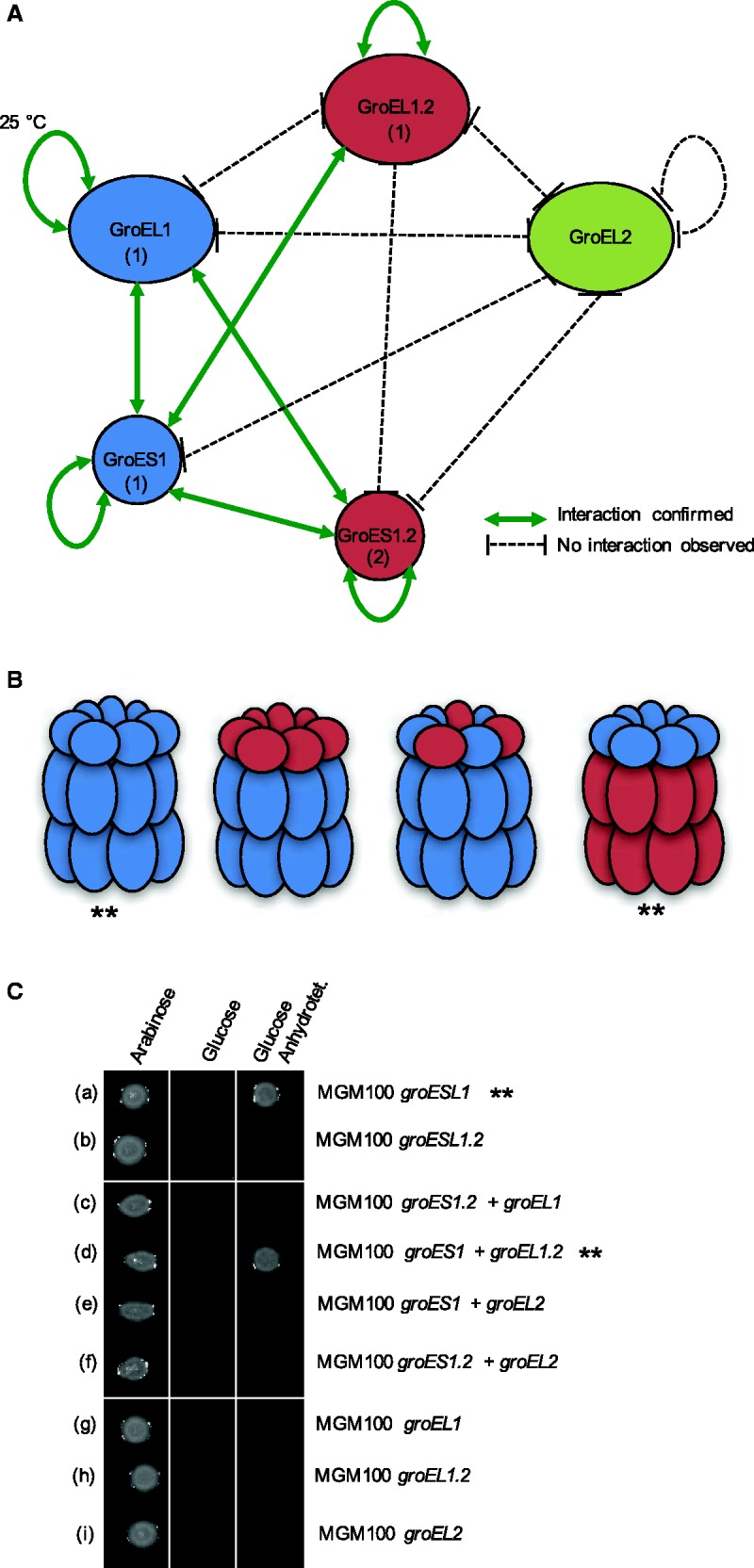
Protein–protein interaction and complementation assay. (*A*) Protein–protein interaction network of GroEL/GroES paralogs in *C. fritschii* PCC 6912. Interaction was tested by bacterial two-hybrid assay with either N- or C- terminally tagged proteins. Screening of positive interaction confirmed by a β-galactosidase assay (supplementary fig. S5, Supplementary Material online). The subunits are marked with (1) if the interaction was observed with both C- and N-terminal tagged proteins or (2) if the interaction was observed only with C-terminal tagged proteins. (*B*) Schematic diagram of putative chaperonin complexes based on the protein–protein interaction network. Chaperonin complexes that perform functional complementation in *E. coli* MGM100 are marked with **. (*C*) Complementation assay in the *groEL* deficient *E. coli* strain MGM100. Plating on arabinose constitutes a positive control (induction of the native *E. coli groESL* operon) whereas plating on glucose constitutes the negative control. Anhydrotetracycline induces the expression of the different cyanobacterial *groES* and *groEL* paralogs (as indicated on the right side). Combinations that compensate the lack of the native *groESL* are marked with **.

The GroES/GroEL chaperonin complex is composed of multiple GroEL subunits that form a barrel-like structure, whereas subunits of GroES form a lid that binds to the GroEL apical domain (Xu et al. 1997). To further study the functional divergence of the paralogs, we examined the structural differences of the three GroEL proteins by testing their ability to form homo- and heteromeric chaperone complexes. Direct interactions between all GroEL and GroES paralogs were tested using a bacterial two-hybrid system with cyclic adenylate cyclase as a split marker. Pairwise interactions of all GroEL and GroES combinations were tested *in vivo* in *E. coli*. All combinations of C- and N-terminally tagged GroES/GroEL pairs were analyzed. Positive interaction was confirmed by β-galactosidase assay (supplementary fig. S5, Supplementary Material online). The results of the interaction assays are summarized in a protein–protein interaction network (fig. 4*A*).

A homomeric interaction was documented for all chaperonin subunits except for GroEL2. Homomeric interaction of GroEL1 paralogs was only detected at low temperature conditions (25 °C), whereas any GroES1.2 protein interaction was only detected when it was C-terminally tagged. We could not detect an interaction between the different GroEL paralogs. Our results so far indicate that GroEL1 and GroEL1.2 form homogeneous barrel-like structures, while GroEL2 functions probably as a monomer. In contrast, the two small subunits, GroES1 and GroES1.2, were found to interact; hence, it is possible that they can form a heterogeneous lid structure. Moreover, we found GroEL1 and GroES1 to interact; this indicates that the *groESL1* encoded proteins can form a chaperonin complex. In contrast, no interaction could be observed between GroEL1.2 and its co-chaperonin GroES1.2, indicating that the *groESL1.2* encoded proteins are not forming a chaperonin complex. In addition, the interaction between GroEL1 and GroES1.2, and GroES1 and GroES1.2 suggests that proteins of the two operons can form different types of hybrid GroESL complexes. One type in which GroEL1 forms the barrel and GroES1 and GroES1.2 form a hybrid lid. A second type comprises a GroEL1 barrel and a GroES1.2 lid. The interaction we found between GroEL1.2 and GroES1 suggests that these two paralogs can form an additional hybrid chaperone complex (fig. 4*B*). The absence of interaction between GroEL2 and any other tested proteins, suggests that GroEL2 does not form a typical chaperonin complex.

### GroEL/GroES Complementation in *E. coli*

To further characterize the functional diversification of the GroES/GroEL paralogs, we performed a complementation assay in *E. coli* MGM100, in which the expression of the native *groES/groEL* operon is controlled by an arabinose-inducible promoter (McLennan and Masters 1998). To this end, we cloned *groES/groEL* combinations into compatible expression vectors, under the control of the anhydrotetracycline-inducible promoter (*Ptet*), and introduced them into *E. coli* MGM100. The ability of the introduced *groES/groEL* combination to complement the native *E. coli groESL* operon was scored positive if colonies were able to form upon induction with anhydrotetracycline. Using this approach we tested the complementation potential of the native *C. fritschii* operons as well as all GroES/GroEL combinations. In addition, because GroEL was shown to refold thermally denatured substrates independently of GroES in *Anabaena* sp. L31 (Potnis et al. 2016), the complementation potential of all GroEL paralogs was tested in the absence of GroES.

Our results revealed that GroES1/GroEL1 can successfully complement the native *E. coli* chaperonin, whereas GroES1.2/GroEL1.2 cannot (fig. 4*C*). This result is in agreement with the lack of interaction between the GroES1.2 and GroEL1.2. From the observed hybrid chaperone complexes only the GroES1/GroEL1.2 combination was found to complement the native *E. coli* chaperonin (fig. 4*C*). This result is consistent with the observed interaction between GroES1 and GroEL2 but not with the observed interaction between GroES1.2 and GroEL1. The expression of GroEL2 in all combinations could not complement the *E. coli* chaperonin. This supports the notion that GroEL2 does not function as a typical chaperonin. None of the *C. fritschii* GroEL paralogs is able to complement the native *E. coli groESL* operon in the absence of a GroES co-chaperonin (fig. 4*C*). A recent study revealed that *groEL1* encoded in *Anabaena sp*. L-31 is able to compensate for the lack of *groESL* in *E. coli* cultured in 42 °C (Potnis et al. 2016). We note, however, that *Anabaena sp*. L-31 encodes only *groESL1* and *groEL2*, hence our result could be explained by the functional diversification of *groESL1* and *groESL1*.2 in Stigonematales.

## Discussion

The phylogenetic reconstruction of *groEL* and *groES* evolutionary history indicates that the ancestor of heterocystous cyanobacteria (Nostocales and Stigonematales) possessed a duplicated *groESL* operon. Furthermore, the comparison of transcriptional regulation, chaperonin assembly and function among *groESL1* and *groESL1.2* paralogs in the stigonematalean cyanobacterium *C. fritschii* PCC 6912 demonstrates that they are functionally diverged. In those species where *groEL* diversification has been studied, frequently one *groEL* copy has maintained the housekeeping function while other paralogs have undergone sub- or neo-functionalization. For example, the genome of *Sinorhizobium meliloti*, an alphaproteobacterium that is often found in symbiosis with legumes, encodes a total of five *groEL* paralogs. Only one paralog (*groEL1*) serves the housekeeping function and is also essential for the symbiotic interaction with plants and the establishment of nodules in the root (Bittner et al. 2007). A second paralog (*groEL2*) can substitute the housekeeping gene but its lower expression level is insufficient for the establishment of symbiosis in *S. meliloti* (Bittner et al. 2007). Earlier studies of *groEL* paralogs in various cyanobacteria indicate that GroESL1 comprise the housekeeping chaperonin (Glatz et al. 1997; Tanaka et al. 1997; Kovacs et al. 2001). Our results demonstrate that the housekeeping function of the chaperonin encoded by *groESL1* is also maintained in *C. fritschii* PCC 6912.

The transcription level of *groESL1.2* varies from that of *groESL1* under standard and different growth conditions. A comparison of the 5′-UTR of the two operons revealed variation in the position and sequence of the regulatory elements (supplementary table S2, Supplementary Material online). Yet, the amino acid sequence similarity between the two chaperonins is high (GroEL1 vs. GroEL1.2: 87.2%; GroES1 vs. GroES1.2: 73.4% identical amino acids), hence, the mode of action is probably not different. This is supported by the ability of GroEL1.2 to complement a *groESL* deficient *E. coli* strain. Nevertheless, the lack of evidence for interaction between GroES1.2 and GroEL1.2 suggests that the *groESL1.2* operon is dysfunctional, in a sense that its members do not interact, although transcribed together (ΔCT Ratio 1.20; supplementary table S4, Supplementary Material online). We note that *groESL1.2* evolves under purifying selection (fig. 1*D*) hence nonfunctionalization of that operon can be ruled out. One reason for the lack of GroES1.2–GroEL1.2 interaction may be due to amino acid replacements at the GroES–GroEL binding sites. However, the known GroES–GroEL interface amino acids (Xu et al. 1997) are conserved among the paralogs in *C. fritschii*. Furthermore, both subunits of the second operon can interact with GroES1, and even complement the *E. coli* chaperonin as shown in the case of GroES1–GroEL1.2. We note that oligomerization of GroEL1 was observed only at 25 °C while GroEL1.2 oligomers could be validated at 30 °C. Additionally, *groEL1.2* transcription is upregulated under high temperature to a higher extent in comparison to *groEL1*. Hence, it is likely that heterologous GroEL1.2–GroES1 chaperonin complexes can be formed in *C. fritschii* and contributes to the high temperature tolerance. The upregulation of *groEL1.2* transcription and the strong promoter activity of both *groESL1* and *groESL1.2* during diazotrophic condition suggest a functional role of the two *groESL* paralogs during heterocyst differentiation, albeit in different stages thereof.

Heterologous chaperonin complexes may also have divergent substrate sets. For example, a study of *groESL* paralogs in the deltaproteobacterium *Myxococcus xanthus* DK1622 reported that the functional diversification of the two duplicates involved a diversification of their substrate sets. Only 35% of the GroEL clients interact with both paralogs in that organism (Wang et al. 2013). The deletion of either one of the paralogs resulted in significantly different development, predation and heat-shock response phenotypes. Hence, the division of client sets between the two paralogs in *M. xanthus* has prominent functional consequences (Wang et al. 2013). Thus, it is possible that the heterologous chaperonin complexes in *C. fritschii* might also have divergent substrate sets. We note that GroES1.2 in combination with either GroEL1.2 or GroEL1 could not complement the *E. coli* chaperonin, which could be due to a different substrate set of that chaperonin complex. Yet, an interaction between GroES1.2 with GroES1 and GroES1.2 with GroEL1 is observed. This raises the possibility that the retention *of groES1.2* has a dose effect by elevating the number of GroES subunits required to form the chaperonin lid. Overall, our study suggests that the retention of *groESL1.2* in Stigonematales is accompanied by a subfunctionalization of that operon.

Our phylogenetic analysis suggests that the duplication of the monocistronic *groEL2* is ancient and most probably was already present in the ancestor of cyanobacteria. The ubiquitous distribution of *groEL2* orthologs in cyanobacteria and the differential regulation under light stress in *C. fritschii* and other cyanobacteria (Glatz et al. 1997) suggest that its function is related to photosynthesis. Our study furthermore shows that GroEL2 cannot form an oligomer (i.e., the typical chaperonin barrel structure) and cannot complement the *E. coli* chaperonin. Thus the GroEL2 mode of action is distinct from that of GroEL1 and GroEL1.2. We conclude that *groEL2* has undergone a neofunctionalization in cyanobacteria.

In summary, our study adds evidence to the hypothesis that *groES* and *groEL* duplicates can be retained during prokaryote evolution and evolve new or modified functions in the cell. Prominent examples of *groESL* subfunctionalization are found in bacterial organisms whose lifestyle includes several developmental stages such as *M. xanthus* (Wang et al. 2013) or lifestyles as in *S. meliloti* (Bittner et al. 2007) and *Mycobacterium smegmatis* (Rao and Lund 2010). Here, we show that evolution of heterocyst differentiation in cyanobacteria was accompanied by *groESL* duplication and diversification of the paralogs.

## Materials and Methods

### Phylogenetic Analysis

The search for GroES/GroEL homologs was performed with BLAST+ (Camacho et al. 2009) using the amino acid sequence of GroES and GroEL encoded in *Escherichia coli* K12 MG1655 as a query. The search database included all sequenced cyanobacterial genomes in NCBI RefSeq (ver. July 2014) (Tatusova et al. 2014) and Joint Genome Institute (JGI; Grigoriev et al. 2012). An *e*-value <10^−^^10^ was used as a sequence similarity threshold. Annotations of the BLAST hits were determined manually according to RefSeq database. Hits that lacked an annotation were validated by reBLASTing against NCBI. Multiple sequence alignments of the GroES/GroEL orthologs were computed with MAFFT ver. 7.027b (Katoh and Standley 2013). Phylogenetic trees were reconstructed using PhyML ver. 3.1 with the Le-Gascuel (LG) substitution model (Le and Gascuel 2008) for amino acid sequences and general time-reversible (GTR) substitution model (Lanave et al. 1984) for nucleotide sequences and SPR search algorithm (Guindon et al. 2010). Testing hypotheses regarding the tree topology was performed by reconstructing a maximum-likelihood tree with a user tree constraint. The comparison of ML phylogeny and the constrained phylogeny was performed using CONSEL ver. 1.2 (Shimodaira and Hasegawa 2001). The calculation of synonymous (dS) and nonsynonymous (dN) substitution rates was performed with CodeML (Yang 2007). Codon alignments for the dN/dS calculation were prepared with PAL2NAL (Suyama et al. 2006). Phylogenetic trees were plotted with FigTree (http://tree.bio.ed.ac.uk/software/figtree/).

### Strains, Culture Conditions and Standard Techniques

*Chlorogloeopsis fritschii PCC 6912* was obtained from the Pasteur Culture Collection (PCC) of cyanobacteria. Stock cultures were grown photoautotrophically in liquid BG11 medium supplemented with or without (BG11o) combined nitrogen at 37 °C and a light intensity of 24 µE m^−^^2^ s^−^^1^ (Rippka et al. 1979). Transformation of *C. fritschii* was performed by triparental mating (Stucken et al. 2012) with selection on 1% agarose BG11 plates supplemented with neomycin (Nm) 30 µg ml^−^^1^. *E. coli* strains XL1 blue and HB101 were used for cloning and conjugation, *E. coli* MGM100 (McLennan and Masters 1998) for complementation and *E. coli* BHT101 for two-hybrid assays. *Escherichia coli* MGM100 was transformed by electroporation. If not otherwise stated, all *E. coli* strains were grown in LB or LB agar. Kanamycin (Km) 15 and 50 µg ml^−^^1^, ampicillin (Amp) 100 µg ml^−^^1^, or spectinomycin (Sp) 50 µg ml^−^^1^ were used for selection.

### DNA Techniques

Genomic DNA was isolated from *C. fritschii* PCC 6912 grown at 37 °C in BG11 medium with orbital shaking (100 rpm). A 30-ml of mid exponentially growing *C. fritschii* cells were harvested by centrifugation and DNA-Isolation was performed as described in (Franche and Damerval 1988), followed by removal of residual RNA with RNase A. PCRs were carried out with Phusion™ polymerase (Thermo Scientific, Germany) following the guidelines of the manufacturer. All primers used in this study are listed in supplementary table S3, Supplementary Material online.

### RNA Isolation and Quantitative Real-Time PCR (qRT-PCR)

Samples for qRT-PCR were harvested by filtration before and 5, 15, and 30 min after induction of high light (70 µE m^−^^2^ s^−^^1^) and high temperature (50 °C) growth conditions. Samples from diazotrophic conditions were harvested 3, 8, and 24 h after nitrogen deprivation. Upon harvesting samples were frozen in liquid nitrogen. Total RNA was isolated using Concert Plant RNA Reagent (Invitrogen, Germany) according to the manufacturer’s instruction and treated with RNase-free DNaseI™ (Ambion, Germany). One microgram of total RNA was used for single-strand cDNA synthesis with iScript™ (Biorad, Germany). Quantitative RT-PCR was performed with primers listed in supplementary table S3, Supplementary Material online in a StepOne Real-Time PCR System (Biorad) with Power SYBR Green PCR master mix (Applied Biosystems, Germany) and 50 ng cDNA as template. All samples were run in biological and technical triplicates. The transcript levels of *groEL* paralogous genes were normalized using housekeeping gene *rnpB* as a reference. Relative transcript levels were calculated using the ΔΔC_t_ method (Livak and Schmittgen 2001). Absolute transcript levels were calculated as described in (Lu et al. 2012).

### Fluorescence Microscopy and Transcriptional Fusions

Samples of the cyanobacterial cultures for fluorescence microscopy were taken before and after 10 and 20 min after the cultures were transferred to high-light conditions (140 µE m^−^^2^ s^−^^1^) and high temperature conditions (50 °C), respectively. Samples from diazotrophic conditions were documented before and every 12 h after nitrogen deprivation over a period of 5 days. To fuse the promoter of *groEL* and *groESL* operons (*P_groE_*) to *gfp:mut3.1*, PstI and BamHI restriction sites were introduced to 5′ and 3′ ends of *gfp-mut3.1* (amplified from pRL153-GFP; Tolonen et al. 2006) and cloned in pBluescript SK(+). EcoRI and PstI sites were introduced to each P_*groE*_ fragment and these were fused upstream to *gfp-mut3.1*. The *P_groE_:gfp-mut3.1* fusions were excised from pBSK+ with EcoRI and BamHI and cloned in the cyanobacterial shuttle vector pRL25C (Wolk et al. 1988).

For time-lapse imaging, gene frame cover slips (Thermo Scientific) were attached to glass slides and the resulting chamber filled with 150 µl of BG11o medium containing agarose (0.5%) and supplemented with neomycin (30 µg ml^−^^1^). To ensure aeration, 0.5 mm slices of solid media were removed from each side of the chamber. Twenty microliters of cells were then placed on top of the agarose slice and the chamber was sealed with a glass coverslip and visualized with an epifluorescence microscope (Zeiss Axio Imager 2, Plan-Apochromat 63×/1.40 Oil DIC M27 objective).

### Protein–Protein Interaction Network

To characterize the physical association between components of the *C. fritschii* GroESL chaperonin complex, proteins were tested systematically for pairwise interaction by using the Bacterial Adenylate Cyclase Two-Hybrid System Kit (BACTH System Kit; Karimova et al. 1998). Both C- and N-termini tags were tested for all paralogs. PstI and BamHI sites were introduced by PCR to all *groES* and *groEL* genes. DNA fragments were digested with PstI and BamHI and cloned into the corresponding sites of the different BACTH plasmids. Screening for the ability to interact was performed according to the protocol on LB agar plates supplemented with X-gal (40 µg ml^−^^1^), IPTG (0.5 mM), Amp and Km. Blue colonies indicated positive interaction and the expression of the reporter gene was subsequently confirmed by β-galactosidase assay (supplementary fig. S4, Supplementary Material online).

### Complementation Assays

Complementation assays were performed with *E. coli* MGM100 expressing *groESL* under the regulation of the arabinose (0.2%) inducible pBAD promoter (McLennan and Masters 1998). The two operons (*groESL1* and *groESL1.2*) and all chaperon subunit encoding genes (*groEL1*, *groEL1.2*, *groEL2*, *groES1*, and *groES1.2*) of *C. fritschii* PCC 6912 were amplified using the primers listed in supplementary table S3, Supplementary Material online. DNA fragments were cloned into pASK-IBA3plus (iba-lifesciences, Göttingen, Germany) or pASK-IBA3sp, an *aadA* (Sp^R^) cassette marker containing derivative of pASK-IBA3plus, using BsaI cutsites. Expression of the proteins was regulated by anhydrotetracycline treatment (0.2 µg ml^−^^1^, 3 h). For MGM100 co-expressing *C. fritschii groEL* and *groES* paralogs from different plasmids, both plasmids were co-transformed by electroporation. Transformed MGM100 was selected on LB plates containing Km, arabinose, Amp or Km, arabinose, Amp, Sp (for co-expression), and cells were grown in liquid media over night. Serially diluted cells were spotted onto selective solidified LB and grown at 30 °C for 18 hours. Growth of *E. coli* MGM100 on plates supplemented with glucose (and anhydrotetracycline) occurred only if the expressed *groES/groEL* from *C. fritschii* can complement the *E. coli* MGM100 strain.

## Supplementary Material

Supplementary data are available at *Genome Biology and Evolution* online.

## Supplementary Material

Supplementary Data

## References

[evw287-B1] Amoros-Moya D Bedhomme S Hermann M Bravo IG. 2010 Evolution in regulatory regions rapidly compensates the cost of nonoptimal codon usage. Mol Biol Evol. 27:2141–2151. 20403964 10.1093/molbev/msq103

[evw287-B2] Bittner AN Foltz A Oke V. 2007 Only one of five *groEL* genes is required for viability and successful symbiosis in *Sinorhizobium meliloti* . J Bacteriol. 189:1884–1889. 17158666 10.1128/JB.01542-06 PMC1855696

[evw287-B3] Bogumil D Dagan T. 2010 Chaperonin-dependent accelerated substitution rates in prokaryotes. Genome Biol Evol. 2:602–608. 20660111 10.1093/gbe/evq044 PMC3296371

[evw287-B4] Bratlie MS, 2010 Gene duplications in prokaryotes can be associated with environmental adaptation. BMC Genomics 11:588. 20961426 10.1186/1471-2164-11-588 PMC3091735

[evw287-B5] Camacho C, 2009 BLAST+: architecture and applications. BMC Bioinform. 10:421. 10.1186/1471-2105-10-421 PMC2803857 20003500

[evw287-B6] Chaurasia AK Apte SK. 2009 Overexpression of the *groESL* operon enhances the heat and salinity stress tolerance of the nitrogen-fixing cyanobacterium *Anabaena* sp. strain PCC7120. Appl Environ Microbiol. 75:6008–6012. 19633117 10.1128/AEM.00838-09 PMC2747873

[evw287-B7] Dagan T, 2013 Genomes of Stigonematalean Cyanobacteria (Subsection V) and the Evolution of Oxygenic Photosynthesis from prokaryotes to plastids. Genome Biol Evol. 5:31–44. 23221676 10.1093/gbe/evs117 PMC3595030

[evw287-B8] Flores E Herrero A. 2010 Compartmentalized function through cell differentiation in filamentous cyanobacteria. Nat Rev Microbiol. 8:39–50. 19966815 10.1038/nrmicro2242

[evw287-B300] Franche C Damerval T. 1988 Test on nif probes and DNA hybridizations. Methods Enzymol. 167:803–808.

[evw287-B9] Fuentes-Hernandez A, 2015 Using a sequential regimen to eliminate bacteria at sublethal antibiotic dosages. PLoS Biol. 13:e1002104. 25853342 10.1371/journal.pbio.1002104 PMC4390231

[evw287-B10] Furuki M Tanaka N Hiyama T Nakamoto H. 1996 Cloning, characterization and functional analysis of *groEL*-like gene from thermophilic cyanobacterium *Synechococcus vulcanus*, which does not form an operon with *groES* . Biochim Biophys Acta. 1294:106–110. 8645726 10.1016/0167-4838(96)00037-4

[evw287-B11] Glatz A, 1997 Chaperonin genes of the *Synechocystis* PCC 6803 are differentially regulated under light-dark transition during heat stress. Biochem Biophys Res Commun. 239:291–297. 9345313 10.1006/bbrc.1997.7463

[evw287-B12] Grigoriev IV, 2012 The genome portal of the Department of Energy Joint Genome Institute. Nucleic Acids Res. 40:D26–D32. 22110030 10.1093/nar/gkr947 PMC3245080

[evw287-B13] Guindon S, 2010 New algorithms and methods to estimate maximum-likelihood phylogenies: assessing the performance of PhyML 3.0. Syst Biol. 59:307–321. 20525638 10.1093/sysbio/syq010

[evw287-B14] Horwich AL Low KB Fenton WA Hirshfield IN Furtak K. 1993 Folding in vivo of bacterial cytoplasmic proteins: role of GroEL. Cell 74:909–917. 8104102 10.1016/0092-8674(93)90470-b

[evw287-B15] Huq S Sueoka K Narumi S Arisaka F Nakamoto H. 2010 Comparative biochemical characterization of two GroEL homologs from the Cyanobacterium *Synechococcus elongatus* PCC 7942. Biosci Biotechnol Biochem. 74:2273–2280. 21071850 10.1271/bbb.100493

[evw287-B16] Jacob F. 1977 Evolution and tinkering. Science 196:1161–1166. 860134 10.1126/science.860134

[evw287-B17] Karimova G Pidoux J Ullmann A Ladant D. 1998 A bacterial two-hybrid system based on a reconstituted signal transduction pathway. Proc Natl Acad Sci U S A. 95:5752–5756. 9576956 10.1073/pnas.95.10.5752 PMC20451

[evw287-B18] Katoh K Standley DM. 2013 MAFFT multiple sequence alignment software version 7: improvements in performance and usability. Mol Biol Evol. 30:772–780. 23329690 10.1093/molbev/mst010 PMC3603318

[evw287-B19] Kojima K Nakamoto H. 2007 A novel light- and heat-responsive regulation of the *groE* transcription in the absence of HrcA or CIRCE in cyanobacteria. FEBS Lett. 581:1871–1880. 17434494 10.1016/j.febslet.2007.03.084

[evw287-B20] Kovács E, 2001 The chaperonins of *Synechocystis* PCC 6803 differ in heat inducibility and chaperone activity. Biochem Biophys Res Commun. 289:908–915. 11735133 10.1006/bbrc.2001.6083

[evw287-B21] Lanave C Preparata G Saccone C Serio G. 1984 A new method for calculating evolutionary substitution rates. J Mol Evol. 20:86–93. 6429346 10.1007/BF02101990

[evw287-B22] Le SQ Gascuel O. 2008 An improved general amino acid replacement matrix. Mol Biol Evol. 25:1307–1320. 18367465 10.1093/molbev/msn067

[evw287-B23] Livak KJ Schmittgen TD. 2001 Analysis of Relative gene expression data using real-time quantitative PCR and the 2^−ΔΔC^T method. Methods 25:402–408. 11846609 10.1006/meth.2001.1262

[evw287-B24] Lu Y Xie L Chen J. 2012 A novel procedure for absolute real-time quantification of gene expression patterns. Plant Methods 8:9. 22404915 10.1186/1746-4811-8-9 PMC3323441

[evw287-B25] Lund PA. 2009 Multiple chaperonins in bacteria – why so many? FEMS Microbiol Rev. 33:785–800. 19416363 10.1111/j.1574-6976.2009.00178.x

[evw287-B26] Lynch M. 2007 The Origins of Genome Architecture. Sunderland (MA): Sinauer Associates.

[evw287-B27] McLennan N Masters M. 1998 GroE is vital for cell-wall synthesis. Nature 392:139–139. 9515958 10.1038/32317

[evw287-B28] Moyers BA Zhang J. 2016 Evaluating phylostratigraphic evidence for widespread de novo gene birth in genome evolution. Mol Biol Evol. 33:1245–1256. 26758516 10.1093/molbev/msw008 PMC5010002

[evw287-B29] Mulkidjanian AY, 2006 The cyanobacterial genome core and the origin of photosynthesis. Proc Natl Acad Sci U S A. 103:13126–13131. 16924101 10.1073/pnas.0605709103 PMC1551899

[evw287-B30] Ohno S. 1970 Evolution by Gene Duplication. Berlin, Heidelberg: Springer Science & Business Media

[evw287-B31] Oren Y, 2014 Transfer of noncoding DNA drives regulatory rewiring in bacteria. Proc Natl Acad Sci U S A. 111:16112–16117. 25313052 10.1073/pnas.1413272111 PMC4234569

[evw287-B32] Potnis AA Rajaram H Apte SK. 2016 GroEL of the nitrogen-fixing cyanobacterium *Anabaena* sp. strain L-31 exhibits GroES and ATP-independent refolding activity. J Biochem. 159:295–304. 26449235 10.1093/jb/mvv100

[evw287-B33] Priya S, 2013 GroEL and CCT are catalytic unfoldases mediating out-of-cage polypeptide refolding without ATP. Proc Natl Acad Sci U S A. 110:7199–7204. 23584019 10.1073/pnas.1219867110 PMC3645539

[evw287-B34] Queitsch C Sangster TA Lindquist S. 2002 Hsp90 as a capacitor of phenotypic variation. Nature 417:618–624. 12050657 10.1038/nature749

[evw287-B35] Rajaram H Apte SK. 2008 Nitrogen status and heat-stress-dependent differential expression of the *cpn60* chaperonin gene influences thermotolerance in the cyanobacterium *Anabaena* . Microbiology 154:317–325. 18174150 10.1099/mic.0.2007/011064-0

[evw287-B36] Rao T Lund PA. 2010 Differential expression of the multiple chaperonins of *Mycobacterium smegmatis* . FEMS Microbiol Lett. 310:24–31. 20618852 10.1111/j.1574-6968.2010.02039.x

[evw287-B37] Rippka R Stanier RY Deruelles J Herdman M Waterbury JB. 1979 Generic assignments, strain histories and properties of pure cultures of Cyanobacteria. J Gen Microbiol. 111:1–61.

[evw287-B38] Romero D Palacios R. 1997 Gene amplification and genomic plasticity in prokaryotes. Annu Rev Genet. 31:91–111. 9442891 10.1146/annurev.genet.31.1.91

[evw287-B39] Sabater-Muñoz B, 2015 Fitness trade-offs determine the role of the molecular chaperonin GroEL in buffering mutations. Mol Biol Evol. 32:2681–2693. 26116858 10.1093/molbev/msv144 PMC4576708

[evw287-B40] Sato S Ikeuchi M Nakamoto H. 2008 Expression and function of a *groEL* paralog in the thermophilic cyanobacterium *Thermosynechococcus elongatus* under heat and cold stress. FEBS Lett. 582:3389–3395. 18786533 10.1016/j.febslet.2008.08.034

[evw287-B41] Shimodaira H Hasegawa M. 2001 CONSEL: for assessing the confidence of phylogenetic tree selection. Bioinformatics 17:1246–1247. 11751242 10.1093/bioinformatics/17.12.1246

[evw287-B42] Shimodaira H. 2002 An approximately unbiased test of phylogenetic tree selection. Syst Biol. 51:492–508. 12079646 10.1080/10635150290069913

[evw287-B43] Stucken K Ilhan J Roettger M Dagan T Martin WF. 2012 Transformation and conjugal transfer of foreign genes into the filamentous multicellular Cyanobacteria (Subsection V) *Fischerella* and *Chlorogloeopsis* . Curr Microbiol. 65:552–560. 22833222 10.1007/s00284-012-0193-5

[evw287-B44] Suyama M Torrents D Bork P. 2006 PAL2NAL: robust conversion of protein sequence alignments into the corresponding codon alignments. Nucleic Acids Res. 34:W609–W612. 16845082 10.1093/nar/gkl315 PMC1538804

[evw287-B45] Tanaka N Hiyama T Nakamoto H. 1997 Cloning, characterization and functional analysis of *groESL* operon from thermophilic cyanobacterium *Synechococcus vulcanus* . Biochim Biophys Acta. 1343:335–348. 9434123 10.1016/s0167-4838(97)00159-3

[evw287-B46] Tatusova T Ciufo S Fedorov B O'Neill K Tolstoy I. 2014 RefSeq microbial genomes database: new representation and annotation strategy. Nucleic Acids Res. 42:D553–D559. 24316578 10.1093/nar/gkt1274 PMC3965038

[evw287-B47] Tokuriki N Tawfik DS. 2009 Chaperonin overexpression promotes genetic variation and enzyme evolution. Nature 459:668–673. 19494908 10.1038/nature08009

[evw287-B48] Tolonen AC Liszt GB Hess WR. 2006 Genetic manipulation of *Prochlorococcus* strain MIT9313: green fluorescent protein expression from an RSF1010 plasmid and Tn5 transposition. Appl Environ Microbiol. 72:7607–7613. 17041154 10.1128/AEM.02034-06 PMC1694220

[evw287-B49] Tumer NE Robinson SJ Haselkorn R. 1983 Different promoters for the *Anabaena* glutamine synthetase gene during growth using molecular or fixed nitrogen. Nature 306:337–342.

[evw287-B50] Wang Y, 2013 Mechanisms involved in the functional divergence of duplicated GroEL chaperonins in *Myxococcus xanthus* DK1622. PLoS Genet. 9:e1003306. e1003311. 10.1371/journal.pgen.1003306 PMC3578752 23437010

[evw287-B51] Warnecke T Hurst LD. 2010 GroEL dependency affects codon usage—support for a critical role of misfolding in gene evolution. Mol Syst Biol. 6:340. 20087338 10.1038/msb.2009.94 PMC2824523

[evw287-B52] Webb R Reddy KJ Sherman LA. 1990 Regulation and sequence of the *Synechococcus* sp. strain PCC 7942 *groESL* operon, encoding a cyanobacterial chaperonin. J Bacteriol. 172:5079–5088. 1975581 10.1128/jb.172.9.5079-5088.1990 PMC213165

[evw287-B53] Williams TA Fares MA. 2010 The Effect of chaperonin buffering on protein evolution. Genome Biol Evol. 2:609–619. 20660109 10.1093/gbe/evq045 PMC3296372

[evw287-B54] Wolk CP, 1988 Isolation and complementation of mutants of *Anabaena* sp. strain PCC 7120 unable to grow aerobically on dinitrogen. J Bacteriol. 170:1239–1244. 2830231 10.1128/jb.170.3.1239-1244.1988 PMC210898

[evw287-B55] Xu Z Horwich AL Sigler PB. 1997 The crystal structure of the asymmetric GroEL-GroES-(ADP)7 chaperonin complex. Nature 388:741–750. 9285585 10.1038/41944

[evw287-B56] Yamazawa A Takeyama H Takeda D Matsunaga T. 1999 UV-A-induced expression of GroEL in the UV-A-resistant marine cyanobacterium *Oscillatoria* sp. NKBG 091600. Microbiology 145(4):949–954. 10220174 10.1099/13500872-145-4-949

[evw287-B57] Yang Z. 2007 PAML 4: phylogenetic analysis by maximum likelihood. Mol Biol Evol. 24:1586–1591. 17483113 10.1093/molbev/msm088

