# Supplementary material for: Evolution of Chaperonin Gene Duplication in Stigonematalean Cyanobacteria (Subsection V)
Source: Genome Biol Evol. 2017 Jan 12;9(1):241–52. doi: 10.1093/gbe/evw287 (PMC5381637; doi:10.1093/gbe/evw287)
Supplement: Supplementary Data [file evw287_Supp.pdf]

Evolution of Chaperonin Gene Duplication in Stigonematalean Cyanobacteria (Subsection V)

Julia Weissenbach[1], Judith Ilhan[1], David Bogumil[2], Nils Hülter[1], Karina Stucken[1], Tal Dagan[1]

[1]Institute of General Microbiology, Christian-Albrechts University of Kiel, Am Botanischen Garten 11, 24118 Kiel, Germany. [2]Present address: The Department of Life Sciences & the National Institute for Biotechnology in the Negev, Ben-Gurion University of the Negev, Beer-Sheva 84105, Israel.

Supplementary Data

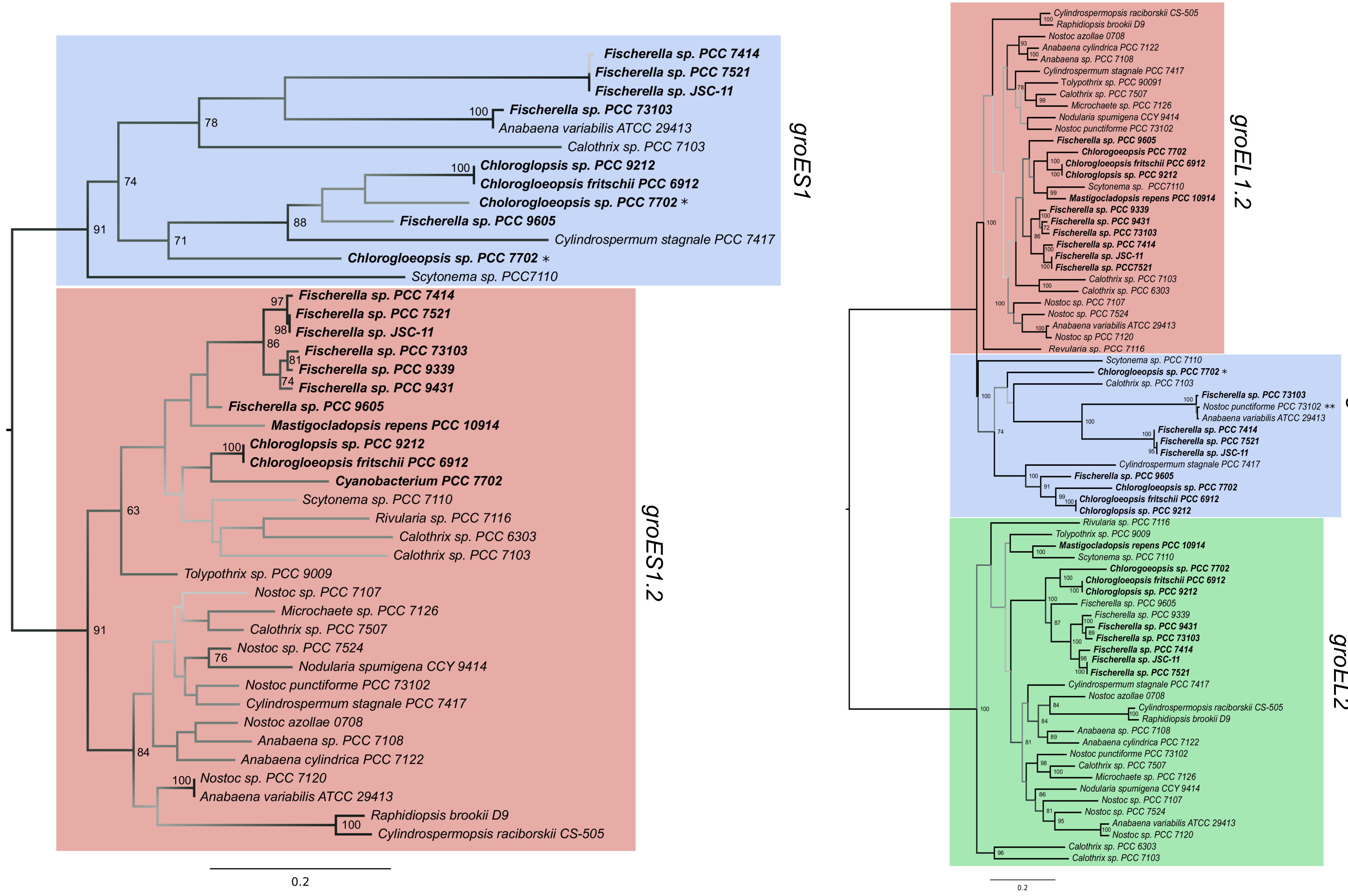

Figure S1. Phylogenetic trees of (A) groES and (B) groEL genes in Nostocales and Stigonematales. Branch color corresponds to bootstrap support, ranging between 5 and 100. Only bootstrap support values >70 are presented.

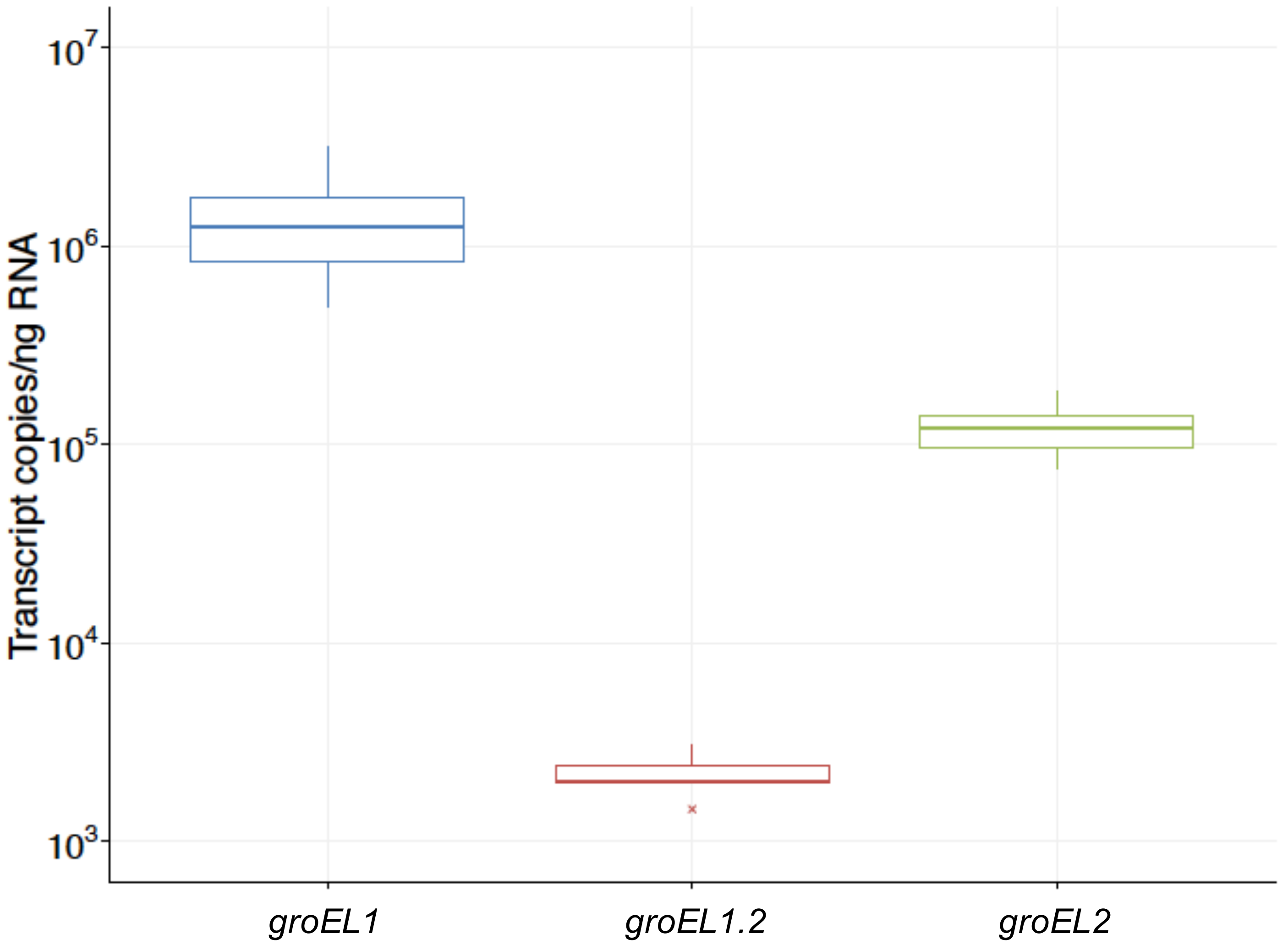

Figure S2: Absolute transcript abundance of *groEL* paralogs in *C. fritschii* PCC 6912 under standard cultivation conditions. RNA was extracted from six independent biological replicates grown under standard conditions.

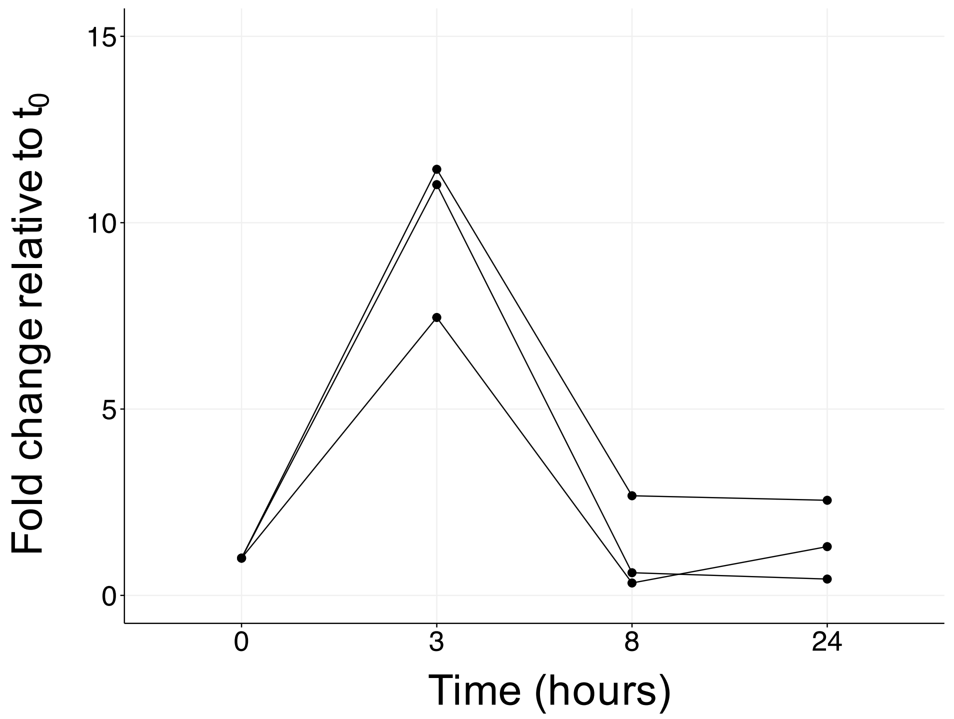

Figure S3. Relative transcript levels of glnA *in C. fritschii* PCC 6912. Graph shows the transcript abundance for three independent biological replicated from cultures grown under daizotrophic conditions. RNA samples were retained before and after *three, eight, and 24 hours of* nitrogen deprivation.

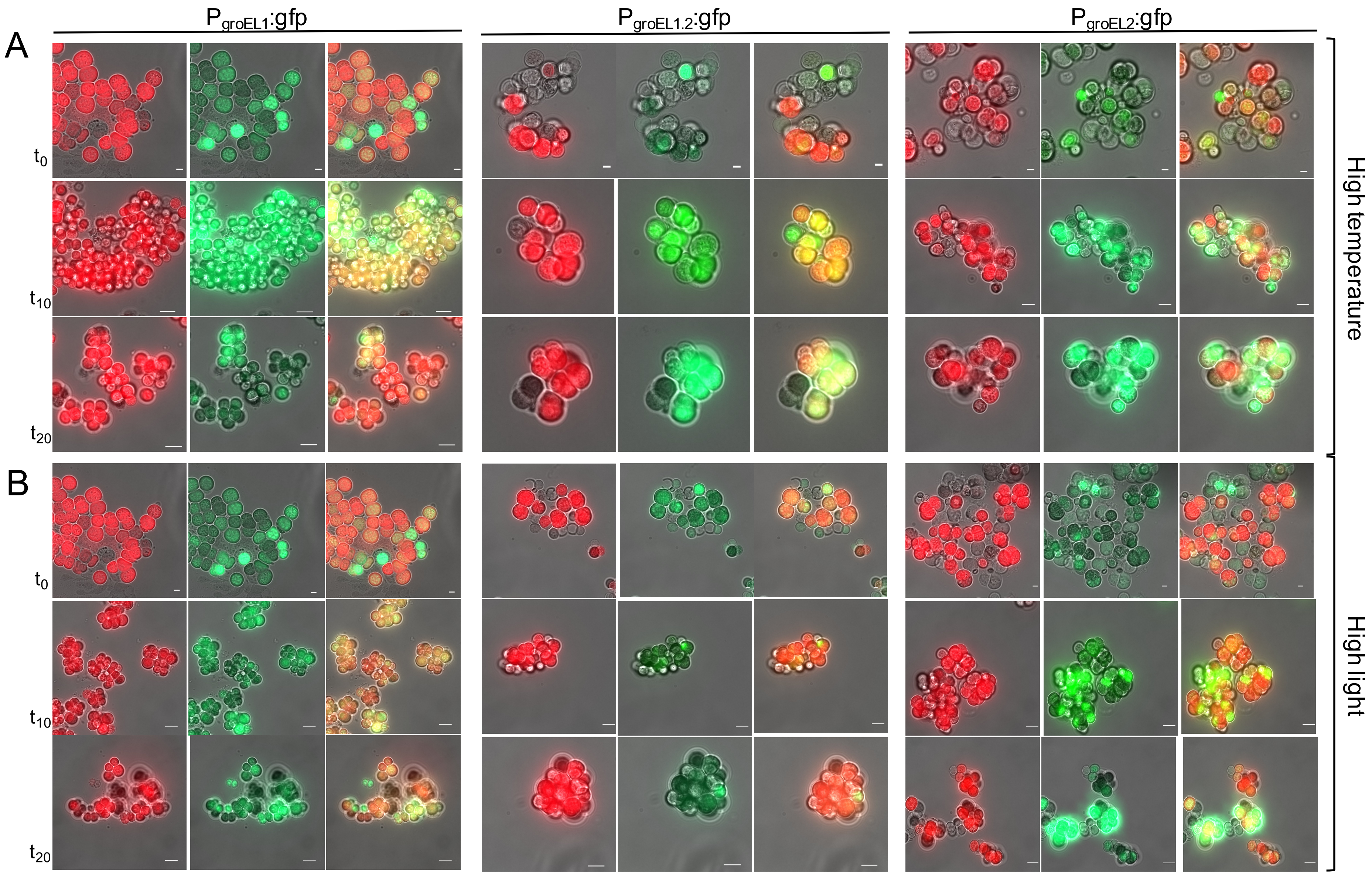

Figure S4. Expression of GFP under control of the different paralogous *groEL* promoters in *C. fritschii* PCC 6912. Merged bright-field plus Chlorophyll fluorescence (red signal); merged bright-field plus GFP fluorescence(green signal) ;and merged bright-field, Chlorophyll-and GFP-flourescence of *C. fritschii* under different growth conditions. (A) High temperature (B) High light. Scale bar represents 10 µm.

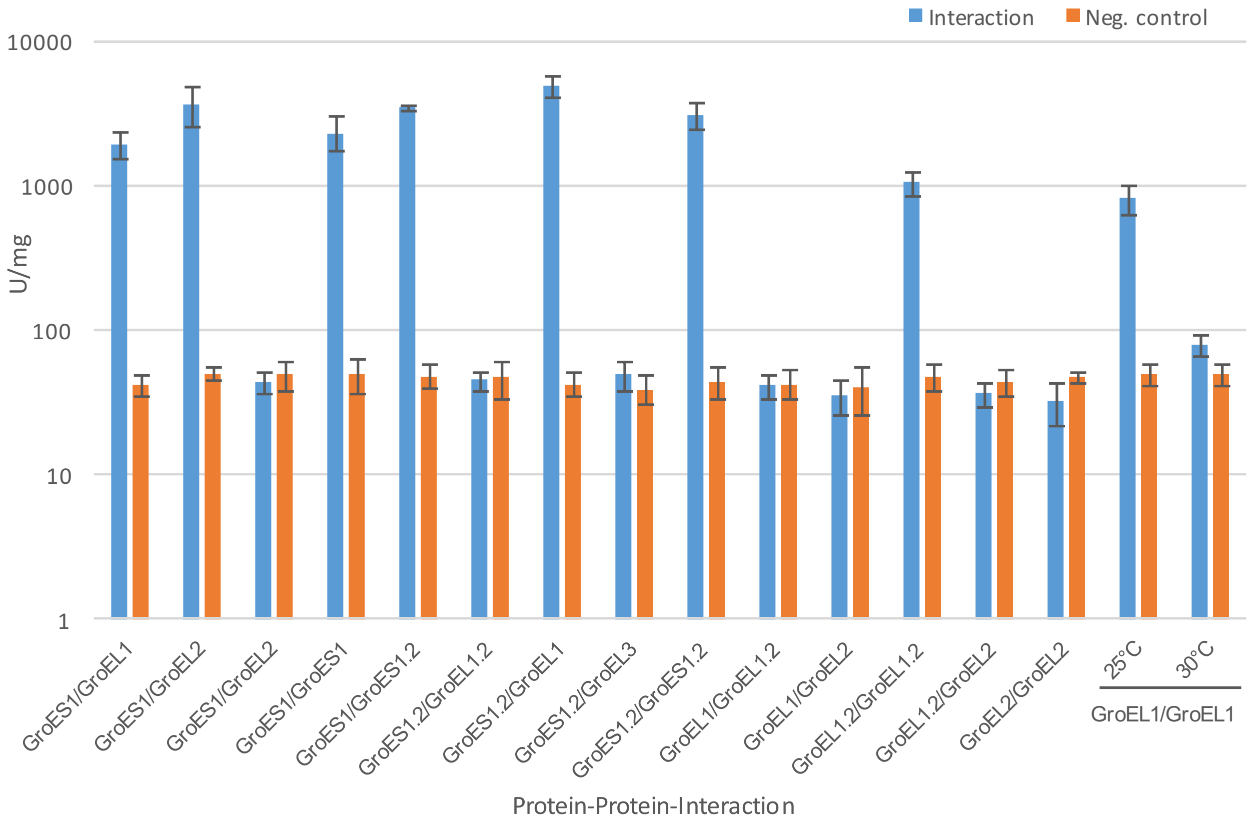

Figure S5. Beta-Galactosidase assay. Bargraphs showing β-galactosidase acivity in the different samples. Lac Z enzyme activity was messured from three indepentent biological replicates.

Table S1. Classification of *groES*/*groEL* genes in cyanobacteria into paralogous gene classes.

Subsection	Organism	Gene	Homolog	Length	RefSeq ID
1	Acaryochloris_marina_MBIC11017	groEL	groEL2	1676	WP_010472301.1
1	Acaryochloris_marina_MBIC11017	groEL	groEL1	1631	WP_012164713.1
1	Acaryochloris_marina_MBIC11017	groES	groEL1	311	WP_010475295.1
1	Acaryochloris_marina_MBIC11017	groES	groES1.2	311	WP_012167682.1
1	Acaryochloris_marina_MBIC11017	groEL	groEL1.2	1613	WP_041661416.1
1	Acaryochloris_sp_CCMEE_5410	groEL	groEL2	1676	WP_010472301.1
1	Acaryochloris_sp_CCMEE_5410	groES	groEL1	311	WP_010475295.1
1	Acaryochloris_sp_CCMEE_5410	groEL	groEL1	1631	WP_010475296.1
1	Acaryochloris_sp_CCMEE_5410	groEL	groEL1.2	1610	WP_010476537.1
1	Acaryochloris_sp_CCMEE_5410	groES	groES1.2	311	WP_010476538.1
1	Chamaesiphon_minutus_PCC_6605	groEL	groEL1	1640	WP_015157755.1
1	Chamaesiphon_minutus_PCC_6605	groES	groEL1	311	WP_015157756.1
1	Chamaesiphon_minutus_PCC_6605	groEL	groEL2	1676	WP_015161862.1
1	Crocosphaera_watsonii_WH_8501	groEL	groEL2	1688	WP_007303269.1
1	Crocosphaera_watsonii_WH_8501	groEL	groEL1	1622	WP_007308122.1
1	Crocosphaera_watsonii_WH_8501	groES	groEL1	311	WP_007308121.1
1	Cyanobacterium_aponinum_PCC_10605	groES	groEL1	311	WP_015218964.1
1	Cyanobacterium_aponinum_PCC_10605	groEL	groEL1	1625	WP_015218965.1
1	Cyanobacterium_aponinum_PCC_10605	groEL	groEL2	1667	WP_015219170.1
1	Cyanobacterium_stanieri_PCC_7202	groEL	groEL2	1664	WP_015221313.1
1	Cyanobacterium_stanieri_PCC_7202	groEL	groEL1	1622	WP_015223059.1
1	Cyanobacterium_stanieri_PCC_7202	groES	groEL1	311	WP_015223060.1
1	cyanobacterium_UCYN-A	groEL	groEL2	1703	WP_012953804.1
1	cyanobacterium_UCYN-A	groES	groEL1	311	WP_012954133.1
1	cyanobacterium_UCYN-A	groEL	groEL1	1628	WP_012954134.1
1	Cyanobium_gracile_PCC_6307	groEL	groEL1	1637	WP_015110666.1
1	Cyanobium_gracile_PCC_6307	groES	groEL1	311	WP_015110667.1
1	Cyanobium_gracile_PCC_6307	groEL	groEL2	1667	WP_015110733.1
1	Cyanobium_sp_PCC_7001	groEL	groEL2	1655	WP_006911651.1
1	Cyanobium_sp_PCC_7001	groES	groEL1	311	WP_006910546.1
1	Cyanobium_sp_PCC_7001	groEL	groEL1	1634	WP_006911407.1
1	Cyanothece_sp_BH63E_ATCC_51472	groES	groEL1	311	WP_008278381.1
1	Cyanothece_sp_BH63E_ATCC_51472	groEL	groEL1	1622	WP_009544164.1
1	Cyanothece_sp_BH63E_ATCC_51472	groEL	groEL2	1679	WP_009545495.1
1	Cyanothece_sp_BH68_ATCC_51142	groES	groEL1	311	WP_008278381.1
1	Cyanothece_sp_BH68_ATCC_51142	groEL	groEL1	1622	WP_009544164.1
1	Cyanothece_sp_BH68_ATCC_51142	groEL	groEL2	1679	WP_009545495.1
1	Cyanothece_sp_CCY_0110	groEL	groEL2	1688	WP_008273166.1
1	Cyanothece_sp_CCY_0110	groEL	groEL1	1622	WP_008278380.1
1	Cyanothece_sp_CCY_0110	groES	groEL1	311	WP_008278381.1
1	Cyanothece_sp_PCC_7424	groEL	groEL1	1628	WP_012599165.1
1	Cyanothece_sp_PCC_7424	groES	groEL1	311	WP_012599166.1
1	Cyanothece_sp_PCC_7424	groEL	groEL2	1670	WP_015954854.1
1	Cyanothece_sp_PCC_7425	groEL	groEL2	1661	WP_012627767.1
1	Cyanothece_sp_PCC_7425	groEL	groEL1	1637	WP_012630459.1
1	Cyanothece_sp_PCC_7425	groES	groEL1	311	WP_012630460.1
1	Cyanothece_sp_PCC_7822	groES	groEL1	311	WP_013320552.1
1	Cyanothece_sp_PCC_7822	groEL	groEL1	1631	WP_013320553.1
1	Cyanothece_sp_PCC_7822	groEL	groEL2	1664	WP_013324620.1
1	Cyanothece_sp_PCC_8801	groEL	groEL2	1667	WP_012594532.1
1	Cyanothece_sp_PCC_8801	groEL	groEL1	1625	WP_012596119.1
1	Cyanothece_sp_PCC_8801	groES	groEL1	311	WP_012596120.1
1	Cyanothece_sp_PCC_8802	groEL	groEL2	1667	WP_012594532.1
1	Cyanothece_sp_PCC_8802	groES	groEL1	311	WP_012596120.1
1	Cyanothece_sp_PCC_8802	groEL	groEL1	1625	WP_012596119.1
1	Dactylococcopsis_salina_PCC_8305	groES	groEL1	311	WP_015228133.1
1	Dactylococcopsis_salina_PCC_8305	groEL	groEL1	1622	WP_015228134.1
1	Dactylococcopsis_salina_PCC_8305	groEL	groEL2	1679	WP_015230198.1
1	Geminocystis_herdmanii_PCC_6308	groES	groEL1	311	WP_017294973.1
1	Geminocystis_herdmanii_PCC_6308	groEL	groEL1	1625	WP_017294974.1
1	Geminocystis_herdmanii_PCC_6308	groEL	groEL2	1673	WP_017295911.1
1	Gloeobacter_violaceus_PCC_7421	groEL	not determined	1628	NP_923973.1
1	Gloeobacter_violaceus_PCC_7421	groES	not determined	311	NP_923974.1
1	Gloeobacter_violaceus_PCC_7421	groES	not determined	311	NP_925842.1
1	Gloeobacter_violaceus_PCC_7421	groEL	not determined	1661	NP_925843.1
1	Gloeobacter_violaceus_PCC_7421	Thermosome-groEL2ike protein	not determined	1517	NP_926694.1
1	Gloeocapsa_sp_PCC_73106	groES	groEL1	311	WP_006530606.1
1	Gloeocapsa_sp_PCC_73106	groEL	groEL1	1625	WP_006530607.1
1	Gloeocapsa_sp_PCC_73106	groEL	groEL2	1670	WP_006528647.1
1	Gloeocapsa_sp_PCC_7428	groES	groEL1	311	WP_015186660.1
1	Gloeocapsa_sp_PCC_7428	groEL	groEL1	1640	WP_015186661.1
1	Gloeocapsa_sp_PCC_7428	groEL	groEL2	1649	WP_015186725.1
1	Halothece_sp_PCC_7418	groES	groEL1	311	WP_015225431.1
1	Halothece_sp_PCC_7418	groEL	groEL1	1625	WP_015225432.1
1	Halothece_sp_PCC_7418	groEL	groEL2	1670	WP_015225564.1
1	Microcystis_aeruginosa_NIES-843	groEL	groEL2	1673	WP_002759846.1
1	Microcystis_aeruginosa_NIES-843	groES	groEL1	311	WP_002737272.1
1	Microcystis_aeruginosa_NIES-843	groEL	groEL1	1625	WP_012267175.1

1	Prochlorococcus_marinus_AS9601	groEL	groEL2	1745	WP_011817963.1
1	Prochlorococcus_marinus_AS9601	groEL	groEL1	1637	WP_011819051.1
1	Prochlorococcus_marinus_AS9601	groES	groEL1	311	WP_011819052.1
1	Prochlorococcus_marinus_marinus_CCMP_1375	groEL	groEL2	1691	NP_874842.1
1	Prochlorococcus_marinus_marinus_CCMP_1375	groEL	groEL1	1637	NP_875980.1
1	Prochlorococcus_marinus_marinus_CCMP_1375	groES	groEL1	311	NP_875981.1
1	Prochlorococcus_marinus_MIT_9211	groEL	groEL2	1697	WP_012195004.1
1	Prochlorococcus_marinus_MIT_9211	groEL	groEL1	1631	WP_012196107.1
1	Prochlorococcus_marinus_MIT_9211	groES	groEL1	311	WP_012196108.1
1	Prochlorococcus_marinus_MIT_9215	groEL	groEL2	1790	WP_041484443.1
1	Prochlorococcus_marinus_MIT_9215	groEL	groEL1	1637	WP_002805622.1
1	Prochlorococcus_marinus_MIT_9215	groES	groEL1	311	WP_002806275.1
1	Prochlorococcus_marinus_MIT_9301	groEL	groEL2	1745	WP_011862474.1
1	Prochlorococcus_marinus_MIT_9301	groEL	groEL1	1637	WP_011863549.1
1	Prochlorococcus_marinus_MIT_9301	groES	groEL1	311	WP_002806275.1
1	Prochlorococcus_marinus_MIT_9303	groES	groEL1	500	WP_011130818.1
1	Prochlorococcus_marinus_MIT_9303	groEL	groEL1	1634	WP_011825179.1
1	Prochlorococcus_marinus_MIT_9303	groEL	groEL2	1694	WP_011825321.1
1	Prochlorococcus_marinus_MIT_9312	groEL	groEL2	1754	WP_011376011.1
1	Prochlorococcus_marinus_MIT_9312	groEL	groEL1	1637	WP_011377072.1
1	Prochlorococcus_marinus_MIT_9312	groES	groEL1	311	WP_011377073.1
1	Prochlorococcus_marinus_MIT_9313	groEL	groEL2	1700	WP_011130702.1
1	Prochlorococcus_marinus_MIT_9313	groEL	groEL1	1634	WP_011130817.1
1	Prochlorococcus_marinus_MIT_9313	groES	groEL1	311	WP_011130818.1
1	Prochlorococcus_marinus_MIT_9515	groEL	groEL2	1763	WP_011819831.1
1	Prochlorococcus_marinus_MIT_9515	groEL	groEL1	1634	WP_011820917.1
1	Prochlorococcus_marinus_MIT_9515	groES	groEL1	311	WP_011820918.1
1	Prochlorococcus_marinus_MIT9202	groEL	groEL1	1637	WP_002805622.1
1	Prochlorococcus_marinus_MIT9202	groES	groEL1	311	WP_002806275.1
1	Prochlorococcus_marinus_MIT9202	groEL	groEL2	1745	WP_041484443.1
1	Prochlorococcus_marinus_NATL1A	groEL	groEL2	1691	WP_011823251.1
1	Prochlorococcus_marinus_NATL1A	groEL	groEL1	1631	WP_011824379.1
1	Prochlorococcus_marinus_NATL1A	groES	groEL1	311	WP_011295315.1
1	Prochlorococcus_marinus_NATL2A	groEL	groEL1	1631	WP_011295314.1
1	Prochlorococcus_marinus_NATL2A	groES	groEL1	311	WP_011295315.1
1	Prochlorococcus_marinus_NATL2A	groEL	groEL2	1682	WP_011294416.1
1	Prochlorococcus_marinus_pastoris_CCMP_1986	groEL	groEL2	1745	WP_011132088.1
1	Prochlorococcus_marinus_pastoris_CCMP_1986	groEL	groEL1	1634	WP_011133065.1
1	Prochlorococcus_marinus_pastoris_CCMP_1986	groES	groEL1	311	WP_011133066.1
1	Prochlorococcus_sp_CC9311	groEL	groEL2	1661	WP_011620049.1
1	Prochlorococcus_sp_CC9311	groEL	groEL1	1631	WP_011620191.1
1	Prochlorococcus_sp_CC9311	groES	groEL1	311	WP_011620192.1
1	Prochlorococcus_sp_CC9605	groEL	groEL2	1679	WP_011363619.1
1	Prochlorococcus_sp_CC9605	groEL	groEL1	1634	WP_006851861.1
1	Prochlorococcus_sp_CC9605	groES	groEL1	311	WP_011365113.1
1	Prochlorococcus_sp_CC9902	groES	groEL1	311	WP_011359322.1
1	Prochlorococcus_sp_CC9902	groEL	groEL1	1634	WP_009790474.1
1	Prochlorococcus_sp_CC9902	groEL	groEL2	1688	WP_011360511.1
1	Prochlorococcus_sp_WH_7803	groEL	groEL2	1661	WP_011933761.1
1	Prochlorococcus_sp_WH_7803	groEL	groEL1	1634	WP_011933891.1
1	Prochlorococcus_sp_WH_7803	groES	groEL1	311	WP_011933892.1
1	Prochlorococcus_sp_WH8102	groES	groEL1	311	WP_011127384.1
1	Prochlorococcus_sp_WH8102	groEL	groEL1	1634	WP_011127385.1
1	Prochlorococcus_sp_WH8102	groEL	groEL2	1688	WP_011128712.1
1	Synechococcus_elongatus_PCC_6301	groEL	groEL2	1667	WP_011243156.1
1	Synechococcus_elongatus_PCC_6301	groES	groEL1	311	WP_011244098.1
1	Synechococcus_elongatus_PCC_6301	groEL	groEL1	1634	WP_011244099.1
1	Synechococcus_elongatus_PCC_7942	groEL	groEL2	1667	WP_011243156.1
1	Synechococcus_elongatus_PCC_7942	groEL	groEL1	1634	WP_011244099.1
1	Synechococcus_elongatus_PCC_7942	groES	groEL1	311	WP_011244098.1
1	Synechococcus_elongatus_PCC_7942_updateJune2012	groEL	groEL2	1667	WP_011243156.1
1	Synechococcus_elongatus_PCC_7942_updateJune2012	groEL	groEL1	1634	WP_011244099.1
1	Synechococcus_elongatus_PCC_7942_updateJune2012	groES	groEL1	311	WP_011244098.1
1	Synechococcus_sp_BL107	groEL	groEL1	1634	WP_009790474.1
1	Synechococcus_sp_BL107	groES	groEL1	311	WP_009790475.1
1	Synechococcus_sp_BL107	groEL	groEL2	1688	WP_009789173.1
1	Synechococcus_sp_CB0101	groEL	groEL2	1667	WP_010303792.1
1	Synechococcus_sp_CB0101	groEL	groEL1	1631	WP_010310877.1
1	Synechococcus_sp_CB0101	groES	groEL1	311	WP_010310879.1
1	Synechococcus_sp_CB0205	groES	groEL1	311	WP_010315361.1
1	Synechococcus_sp_CB0205	groEL	groEL1	1634	WP_010315362.1
1	Synechococcus_sp_CB0205	groEL	groEL2	1661	WP_010318014.1
1	Synechococcus_sp_CC9616	groEL	groEL2	1715	WP_028951375.1
1	Synechococcus_sp_CC9616	groEL	groEL1	1634	WP_028952751.1
1	Synechococcus_sp_CC9616	groES	groEL1	311	WP_028952752.1
1	Synechococcus_sp_JA-2-3Ba2-13	groEL	groEL1	1634	WP_011433224.1
1	Synechococcus_sp_JA-2-3Ba2-13	groES	groEL1	311	WP_011433225.1
1	Synechococcus_sp_JA-2-3Ba2-13	groEL	groEL2	1619	WP_011433315.1
1	Synechococcus_sp_JA-3-3Ab	groEL	groEL2	1628	WP_011429241.1

1	Synechococcus_sp_JA-3-3Ab	groES	groEL1	311	WP_011430254.1
1	Synechococcus_sp_JA-3-3Ab	groEL	groEL1	1634	WP_011430255.1
1	Synechococcus_sp_PCC_6312	groEL	groEL2	1661	WP_015123389.1
1	Synechococcus_sp_PCC_6312	groES	groEL1	311	WP_015124241.1
1	Synechococcus_sp_PCC_6312	groEL	groEL1	1637	WP_015124242.1
1	Synechococcus_sp_PCC_7002	groEL	groEL2	1667	WP_012305785.1
1	Synechococcus_sp_PCC_7002	groES	groEL1	311	WP_012308053.1
1	Synechococcus_sp_PCC_7002	groEL	groEL1	1625	WP_012308054.1
1	Synechococcus_sp_PCC_7335	groES	groEL1	314	WP_006456873.1
1	Synechococcus_sp_PCC_7335	groEL	groEL1	1571	WP_038015768.1
1	Synechococcus_sp_PCC_7335	groEL	not determined	1679	WP_006456066.1
1	Synechococcus_sp_PCC_7335	groEL	not determined	1625	WP_006457968.1
1	Synechococcus_sp_PCC_7336	groEL	groES1.2	1670	WP_017324774.1
1	Synechococcus_sp_PCC_7336	groES	groEL1.2	311	WP_017324775.1
1	Synechococcus_sp_PCC_7336	groES	groEL1	311	WP_017326109.1
1	Synechococcus_sp_PCC_7336	groEL	groEL1	1628	WP_017326110.1
1	Synechococcus_sp_PE_A1_60AY6Li	groES	groEL1	311	WP_011430254.1
1	Synechococcus_sp_PE_A1_60AY6Li	groEL	groEL1	1634	WP_011430255.1
1	Synechococcus_sp_PE_A1_60AY6Li	groEL	groEL2	1628	WP_011429241.1
1	Synechococcus_sp_PE_A1-1_60AY4M2	groEL	groEL1	1634	WP_011430255.1
1	Synechococcus_sp_PE_A1-1_60AY4M2	groES	groEL1	311	WP_011433225.1
1	Synechococcus_sp_PE_A1-1_60AY4M2	groEL	groEL2	1628	WP_011429241.1
1	Synechococcus_sp_PE_A1-1_63AY4M1	groEL	groEL1	1634	WP_011430255.1
1	Synechococcus_sp_PE_A1-1_63AY4M1	groES	groEL1	311	WP_011430254.1
1	Synechococcus_sp_PE_A1-1_63AY4M1	groEL	groEL2	1628	WP_011429241.1
1	Synechococcus_sp_PE_A1-1_65AY640	groEL	groEL1	1634	WP_011430255.1
1	Synechococcus_sp_PE_A1-1_65AY640	groES	groEL1	311	WP_011433225.1
1	Synechococcus_sp_PE_A1-1_65AY640	groEL	groEL2	1628	WP_011429241.1
1	Synechococcus_sp_PE_A4_65AY6A5	groEL	groEL2	1628	WP_011429241.1
1	Synechococcus_sp_PE_A4_65AY6A5	groES	groEL1	311	WP_011429241.1
1	Synechococcus_sp_PE_A4_65AY6A5	groEL	groEL1	1634	WP_011430255.1
1	Synechococcus_sp_PE_A6_63AY4M2	groEL	groEL1	1634	WP_011430255.1
1	Synechococcus_sp_PE_A6_63AY4M2	groES	groEL1	311	WP_011433225.1
1	Synechococcus_sp_PE_A6_63AY4M2	groEL	groEL2	1628	WP_011429241.1
1	Synechococcus_sp_RCC_307	groEL	groEL2	422	WP_011935674.1
1	Synechococcus_sp_RCC_307	groEL	groEL1	1643	WP_011936275.1
1	Synechococcus_sp_RCC_307	groES	groEL1	368	WP_043737222.1
1	Synechococcus_sp_RS9916	groEL	groEL2	1667	WP_007098631.1
1	Synechococcus_sp_RS9916	groEL	groEL1	1634	WP_007098818.1
1	Synechococcus_sp_RS9916	groES	groEL1	311	WP_007098819.1
1	Synechococcus_sp_RS9917	groES	groEL1	311	WP_007101657.1
1	Synechococcus_sp_RS9917	groEL	groEL1	1634	WP_007101658.1
1	Synechococcus_sp_RS9917	groEL	groEL2	1688	WP_038001879.1
1	Synechococcus_sp_WH_8016	groES	groEL1	311	WP_006854300.1
1	Synechococcus_sp_WH_8016	groEL	groEL1	1631	WP_006854299.1
1	Synechococcus_sp_WH_8016	groEL	groEL2	1661	WP_006853514.1
1	Synechococcus_sp_WH_8109	groES	groEL1	311	WP_006850452.1
1	Synechococcus_sp_WH_8109	groEL	groEL1	1634	WP_006851861.1
1	Synechococcus_sp_WH_8109	groEL	groEL2	1703	WP_025362127.1
1	Synechococcus_sp_WH5701	groEL	groEL1	1634	WP_006171951.1
1	Synechococcus_sp_WH5701	groES	groEL1	311	WP_006171952.1
1	Synechococcus_sp_WH5701	groEL	groEL2	1532	N.A
1	Synechococcus_sp_WH7805	groES	groEL1	311	WP_006043324.1
1	Synechococcus_sp_WH7805	groEL	groEL1	1634	WP_006043325.1
1	Synechococcus_sp_WH7805	groEL	groEL2	1670	WP_006043511.1
1	Synechocystis_sp_PCC_6803	groES	groEL1	320	WP_020861555.1
1	Synechocystis_sp_PCC_6803	groEL	groEL1	1625	WP_010872041.1
1	Synechocystis_sp_PCC_6803	groEL	groEL2	1658	WP_010873466.1
1	Synechocystis_sp_PCC_6803_GT-I	groES	groEL1	320	WP_020861555.1
1	Synechocystis_sp_PCC_6803_GT-I	groEL	groEL1	1625	WP_010872041.1
1	Synechocystis_sp_PCC_6803_GT-I	groEL	groEL2	1658	WP_010873466.1
1	Synechocystis_sp_PCC_6803_GT-S	groES	groEL1	320	WP_020861555.1
1	Synechocystis_sp_PCC_6803_GT-S	groEL	groEL1	1625	WP_010872041.1
1	Synechocystis_sp_PCC_6803_GT-S	groEL	groEL2	1658	WP_010873466.1
1	Synechocystis_sp_PCC_6803_PCC-N	groES	groEL1	320	WP_020861555.1
1	Synechocystis_sp_PCC_6803_PCC-N	groEL	groEL1	1625	WP_010872041.1
1	Synechocystis_sp_PCC_6803_PCC-N	groEL	groEL2	1658	WP_010873466.1
1	Synechocystis_sp_PCC_7509	groEL	groEL2	1664	WP_009632953.1
1	Synechocystis_sp_PCC_7509	groEL	groEL1	1637	WP_009633919.1
1	Synechocystis_sp_PCC_7509	groES	groEL1	311	WP_009633920.1
1	Thermosynechococcus_elongatus_BP-1	groEL	groEL1	1637	NP_680976.1
1	Thermosynechococcus_elongatus_BP-1	groES	groEL1	311	NP_680977.1
1	Thermosynechococcus_elongatus_BP-1	groEL	groEL2	1631	NP_682202.1
2	Chroococcidiopsis_sp_PCC_6712	groES	groEL1	311	N. A (JGI)
2	Chroococcidiopsis_sp_PCC_6712	groEL	groEL1	1625	N. A (JGI)
2	Chroococcidiopsis_sp_PCC_6712	groEL	groEL2	1670	N. A (JGI)
2	Chroococcidiopsis_thermalis_PCC_7203	groEL	groEL2	1667	WP_015152570.1
2	Chroococcidiopsis_thermalis_PCC_7203	groEL	groEL1	1640	WP_015152680.1
2	Chroococcidiopsis_thermalis_PCC_7203	groES	groEL1	335	WP_039713348.1

2	Pleurocapsa_sp_PCC_7319	groEL	groEL2	1679	WP_019504182.1
2	Pleurocapsa_sp_PCC_7319	groEL	groEL1	1625	WP_019506109.1
2	Pleurocapsa_sp_PCC_7319	groES	groEL1	311	WP_019506110.1
2	Pleurocapsa_sp_PCC_7327	groEL	groEL1	1634	WP_015142261.1
2	Pleurocapsa_sp_PCC_7327	groES	groEL1	311	WP_015142262.1
2	Pleurocapsa_sp_PCC_7327	groEL	groEL2	1652	WP_015142786.1
2	Stanieria_cyanosphaera_PCC_7437	groEL	groEL1	1625	WP_015193018.1
2	Stanieria_cyanosphaera_PCC_7437	groES	groEL1	311	WP_015193019.1
2	Stanieria_cyanosphaera_PCC_7437	groEL	groEL2	1664	WP_015195459.1
2	Xenococcus_sp_PCC_7305	groEL	groEL1	1622	WP_006511386.1
2	Xenococcus_sp_PCC_7305	groES	groEL1	311	WP_006511387.1
2	Xenococcus_sp_PCC_7305	groEL	groEL2	1685	WP_006510424.1
3	Arthrospira_maxima_CS-328	groEL	groEL2	1676	WP_006625833.1
3	Arthrospira_maxima_CS-328	groEL	groEL1	1637	WP_006622402.1
3	Arthrospira_maxima_CS-328	groES	groEL1	311	WP_006618364.1
3	Arthrospira_platensis_C1_Draft2_circular__genome	groES	groEL1	311	WP_006618364.1
3	Arthrospira_platensis_C1_Draft2_circular__genome	groEL	groEL1	1637	WP_006622402.1
3	Arthrospira_platensis_C1_Draft2_circular__genome	groEL	groEL2	1676	WP_006625833.1
3	Arthrospira_platensis_NIES-39	groES	groEL1	311	WP_006618364.1
3	Arthrospira_platensis_NIES-39	groEL	groEL1	1637	WP_006618363.1
3	Arthrospira_platensis_NIES-39	groEL	groEL2	1676	WP_014276880.1
3	Arthrospira_platensis_Paraca	groEL	groEL2	1627	WP_014276880.1
3	Arthrospira_platensis_Paraca	groEL	groEL1	1637	WP_006618363.1
3	Arthrospira_platensis_Paraca	groES	groEL1	311	WP_006618364.1
3	Arthrospira_sp_PCC_8005	groEL	groEL2	1676	WP_006625833.1
3	Arthrospira_sp_PCC_8005	groES	groEL1	311	WP_006618364.1
3	Arthrospira_sp_PCC_8005	groEL	groEL1	1637	WP_006622402.1
3	Crinalium_epipsammum_PCC_9333	groEL	groEL2	1676	WP_015201979.1
3	Crinalium_epipsammum_PCC_9333	groEL	groEL1	1637	WP_015202370.1
3	Crinalium_epipsammum_PCC_9333	groES	groEL1	311	WP_015202371.1
3	Cyanobacterium_sp_ESFC-1	groES	groEL1	311	WP_018398606.1
3	Cyanobacterium_sp_ESFC-1	groEL	groEL1	1622	WP_018398607.1
3	Cyanobacterium_sp_ESFC-1	groEL	groEL2	1685	WP_018399554.1
3	Cyanobacterium_sp_JSC-1	groEL	groEL1.2	1628	WP_035999449.1
3	Cyanobacterium_sp_JSC-1	groES	groES1.2	311	WP_035999452.1
3	Cyanobacterium_sp_JSC-1	groEL	groEL2	1676	WP_036001242.1
3	Cyanobacterium_sp_JSC-1	groES	groEL1	311	WP_035999452.1
3	Cyanobacterium_sp_JSC-1	groEL	groEL1	1637	WP_036004427.1
3	Geitlerinema_sp_PCC_7105	groEL	groEL2	1682	WP_017663465.1
3	Geitlerinema_sp_PCC_7105	groES	groEL1	311	WP_026097753.1
3	Geitlerinema_sp_PCC_7105	groEL	groEL1	1637	WP_017661781.1
3	Geitlerinema_sp_PCC_7407	groEL	groEL2	1670	WP_015170801.1
3	Geitlerinema_sp_PCC_7407	groEL	groEL1	1637	WP_015172922.1
3	Geitlerinema_sp_PCC_7407	groES	groEL1	311	WP_015172923.1
3	Leptolyngbya_boryana_PCC_6306	groES	groEL1	311	WP_017286920.1
3	Leptolyngbya_boryana_PCC_6306	groEL	groEL1	1640	WP_026148478.1
3	Leptolyngbya_boryana_PCC_6306	groEL	groEL2	1667	WP_017288542.1
3	Leptolyngbya_sp_PCC_6406	groES	not determined	311	WP_008310159.1
3	Leptolyngbya_sp_PCC_6406	groES	groEL1	311	WP_008312546.1
3	Leptolyngbya_sp_PCC_6406	groEL	groEL1	1637	WP_008312275.1
3	Leptolyngbya_sp_PCC_6406	groEL	groEL2	1748	WP_027269099.1
3	Leptolyngbya_sp_PCC_7375	groEL	not determined	1586	WP_006513616.1
3	Leptolyngbya_sp_PCC_7375	groEL	groEL1.2	1616	WP_006513937.1
3	Leptolyngbya_sp_PCC_7375	groES	groES1.2	311	WP_006513938.1
3	Leptolyngbya_sp_PCC_7375	groEL	not determined	1679	WP_006515196.1
3	Leptolyngbya_sp_PCC_7375	groEL	groEL1	1628	WP_006516273.1
3	Leptolyngbya_sp_PCC_7375	groES	groEL1	311	WP_006516274.1
3	Leptolyngbya_sp_PCC_7376	groEL	groEL2	1679	WP_015134386.1
3	Leptolyngbya_sp_PCC_7376	groEL	groEL1	1625	WP_015135231.1
3	Leptolyngbya_sp_PCC_7376	groES	groEL1	311	WP_015135232.1
3	Lyngbya_sp_CCY_8106	groEL	groEL2	1694	WP_009786498.1
3	Lyngbya_sp_CCY_8106	groES	groEL1	311	WP_009787538.1
3	Lyngbya_sp_CCY_8106	groEL	groEL1	1631	WP_009787539.1
3	Microcoleus_chthonoplastes_PCC_7420	groEL	groEL2	1673	WP_006099811.1
3	Microcoleus_chthonoplastes_PCC_7420	groES	groEL1	311	WP_006101488.1
3	Microcoleus_chthonoplastes_PCC_7420	groEL	groEL1	1628	WP_006101523.1
3	Microcoleus_sp_PCC_7113	groEL	groEL1	1640	WP_015184308.1
3	Microcoleus_sp_PCC_7113	groES	groEL1	311	WP_015184309.1
3	Microcoleus_sp_PCC_7113	groEL	groEL2	1673	WP_015185157.1
3	Nodosilinea_nodulosa_PCC_7104	groEL	groEL2	1673	WP_017296590.1
3	Nodosilinea_nodulosa_PCC_7104	groEL	groEL1.2	1634	WP_017298742.1
3	Nodosilinea_nodulosa_PCC_7104	groES	groES1.2	311	WP_017298743.1
3	Nodosilinea_nodulosa_PCC_7104	groEL	groEL1	1631	WP_026072825.1
3	Nodosilinea_nodulosa_PCC_7104	groES	groEL1	311	WP_017299961.1
3	Oscillatoria_acuminata_PCC_6304	groEL	groEL1	1634	WP_015147151.1
3	Oscillatoria_acuminata_PCC_6304	groES	groEL1	311	WP_015147152.1
3	Oscillatoria_acuminata_PCC_6304	groEL	groEL2	1688	WP_015149515.1
3	Oscillatoria_formosa_PCC_6407	groES	groEL1	311	WP_007353484.1
3	Oscillatoria_formosa_PCC_6407	groEL	groEL1	1640	WP_007353485.1

3	Oscillatoria_formosa_PCC_6407	groEL	groEL2	1688	WP_007353288.1
3	Oscillatoria_nigro-viridis_PCC_7112	groEL	groEL1	1643	WP_015174510.1
3	Oscillatoria_nigro-viridis_PCC_7112	groES	groEL1	311	WP_006633072.1
3	Oscillatoria_nigro-viridis_PCC_7112	groEL	groEL2	1682	WP_015177119.1
3	Oscillatoria_sp_PCC_10802	groEL	groEL2	1667	WP_017715999.1
3	Oscillatoria_sp_PCC_10802	groEL	groEL1	1640	WP_017717869.1
3	Oscillatoria_sp_PCC_10802	groES	groEL1	311	WP_017717870.1
3	Oscillatoria_sp_PCC_6506	groEL	groEL2	1688	WP_007353288.1
3	Oscillatoria_sp_PCC_6506	groES	groEL1	311	WP_007353484.1
3	Oscillatoria_sp_PCC_6506	groEL	groEL1	1640	WP_007353485.1
3	Oscillatoriales_sp_JSC-12	groEL	groEL2	1649	WP_009554131.1
3	Oscillatoriales_sp_JSC-12	groES	groEL2	344	WP_051164050.1
3	Oscillatoriales_sp_JSC-12	groEL	groEL1	1637	WP_009768317.1
3	Oscillatoriales_sp_JSC-12	groES	groES1	311	WP_009768318.1
3	Prochlorothrix_hollandica_PCC_9006	groEL	groEL1	1631	WP_017713500.1
3	Prochlorothrix_hollandica_PCC_9006	groES	groES1	311	WP_016925061.1
3	Prochlorothrix_hollandica_PCC_9006	groEL	groEL2	1679	WP_017711036.1
3	Pseudanabaena_sp_PCC_6802	groES	groES1.2	311	WP_026103227.1
3	Pseudanabaena_sp_PCC_6802	groEL	groEL1.2	1661	WP_019500096.1
3	Pseudanabaena_sp_PCC_6802	groEL	groEL1	1628	WP_019501755.1
3	Pseudanabaena_sp_PCC_6802	groES	groES1	311	WP_019501756.1
3	Pseudanabaena_sp_PCC_7367	groEL	groEL1	1628	WP_015165256.1
3	Pseudanabaena_sp_PCC_7367	groES	groES1	308	WP_015165257.1
3	Pseudanabaena_sp_PCC_7367	groES	groES1	311	WP_015165610.1
3	Pseudanabaena_sp_PCC_7367	groEL	groEL1.2	1664	WP_015165611.1
3	Pseudanabaena_sp_PCC_7429	groEL	groEL1	1634	WP_009629537.1
3	Pseudanabaena_sp_PCC_7429	groES	groES1	311	WP_009629538.1
3	Pseudanabaena_sp_PCC_7429	groEL	groEL1.2	1664	WP_009626159.1
3	Pseudanabaena_sp_PCC_7429	groES	groES1.2	311	WP_009626160.1
3	Spirulina_major_PCC_6313	groES	groES1	311	N. A (JGI)
3	Spirulina_major_PCC_6313	groEL	groEL1	1625	N. A (JGI)
3	Spirulina_major_PCC_6313	groEL	groEL2	1673	N. A (JGI)
3	Spirulina_subsalsa_PCC_9445	groEL	groEL2	1667	WP_017303097.1
3	Spirulina_subsalsa_PCC_9445	groES	groES1	314	WP_026079937.1
3	Spirulina_subsalsa_PCC_9445	groEL	groEL1	1625	WP_017306382.1
3	Trichodesmium_erythraeum_IMS101	groEL	groEL2	1685	WP_011610149.1
3	Trichodesmium_erythraeum_IMS101	groES	groES1	311	WP_011613645.1
3	Trichodesmium_erythraeum_IMS101	groEL	groEL1	1634	WP_011613646.1
4	Anabaena_cylindrica_PCC_7122	groEL	groEL2	1718	WP_015216715.1
4	Anabaena_cylindrica_PCC_7122	groEL	groEL1	1637	WP_015217475.1
4	Anabaena_cylindrica_PCC_7122	groES	groES1	311	WP_015217477.1
4	Anabaena_sp_PCC_7108	groEL	groEL2	1664	WP_016949529.1
4	Anabaena_sp_PCC_7108	groES	groES1	311	WP_016951426.1
4	Anabaena_sp_PCC_7108	groEL	groEL1	1637	WP_016951427.1
4	Anabaena_variabilis_ATCC_29413	groEL	groEL1.2	1637	WP_011316758.1
4	Anabaena_variabilis_ATCC_29413	groES	groES1.2	311	WP_011316757.1
4	Anabaena_variabilis_ATCC_29413	groEL	groEL1	1634	WP_011320337.1
4	Anabaena_variabilis_ATCC_29413	groES	groES1	311	WP_010997805.1
4	Anabaena_variabilis_ATCC_29413	groEL	groEL2	1682	WP_011320465.1
4	Calothrix_sp_PCC_6303	groEL	groEL2	1709	WP_041739258.1
4	Calothrix_sp_PCC_6303	groEL	groEL1	1637	WP_015200995.1
4	Calothrix_sp_PCC_6303	groES	groES1	335	WP_041740016.1
4	Calothrix_sp_PCC_7103	groEL	groEL2	1679	WP_006625833.1
4	Calothrix_sp_PCC_7103	groES	groES1.2	311	WP_019490874.1
4	Calothrix_sp_PCC_7103	groEL	groEL1.2	1628	WP_019490875.1
4	Calothrix_sp_PCC_7103	groES	groES1	314	WP_019491741.1
4	Calothrix_sp_PCC_7103	groEL	groEL1	1640	WP_019491742.1
4	Calothrix_sp_PCC_7507	groEL	groEL2	1679	WP_015127399.1
4	Calothrix_sp_PCC_7507	groES	groES1	311	WP_015130973.1
4	Calothrix_sp_PCC_7507	groEL	groEL1	1634	WP_015130974.1
4	Cylindrospermopsis_raciborskii_CS-505	groES	groES1	311	WP_006278399.1
4	Cylindrospermopsis_raciborskii_CS-505	groEL	groEL1	1637	WP_006278400.1
4	Cylindrospermopsis_raciborskii_CS-505	groEL	groEL2	1685	WP_006278498.1
4	Cylindrospermum_stagnale_PCC_7417	groES	groES1	311	WP_015205864.1
4	Cylindrospermum_stagnale_PCC_7417	groEL	groEL1	1640	WP_015205865.1
4	Cylindrospermum_stagnale_PCC_7417	groES	groES1.2	311	WP_015206484.1
4	Cylindrospermum_stagnale_PCC_7417	groEL	groEL1.2	1631	WP_015206485.1
4	Cylindrospermum_stagnale_PCC_7417	groEL	groEL2	1727	WP_015207554.1
4	Microchaete_sp_PCC_7126	groEL	groEL1	1634	WP_017652696.1
4	Microchaete_sp_PCC_7126	groES	groES1	311	WP_015130973.1
4	Microchaete_sp_PCC_7126	groEL	groEL2	1673	WP_017652866.1
4	Nodularia_spumigena_CCY9414	groEL	groEL1	1637	WP_006194607.1
4	Nodularia_spumigena_CCY9414	groES	groES1	311	WP_006194609.1
4	Nodularia_spumigena_CCY9414	groEL	groEL2	1703	WP_042202155.1
4	Nostoc_azollae_0708	groEL	groEL1	1631	WP_013191732.1
4	Nostoc_azollae_0708	groES	groES1	311	WP_013191733.1
4	Nostoc_azollae_0708	groEL	groEL2	1730	WP_041643235.1
4	Nostoc_punctiforme_PCC_73102	groEL	groEL1	1634	WP_012407589.1
4	Nostoc_punctiforme_PCC_73102	groES	groES1	311	WP_012407590.1

4	Nostoc_punctiforme_PCC_73102	groEL	not determined	1682	WP_012407975.1
4	Nostoc_punctiforme_PCC_73102	groEL	not determined	1637	WP_012409490.1
4	Nostoc_sp_PCC_7107	groEL	groEL2	1664	WP_015111848.1
4	Nostoc_sp_PCC_7107	groES	groES1	311	WP_015113912.1
4	Nostoc_sp_PCC_7107	groEL	groEL1	1637	WP_015113913.1
4	Nostoc_sp_PCC_7120	groEL	groEL2	1682	WP_010996060.1
4	Nostoc_sp_PCC_7120	groES	groES1	311	WP_010997805.1
4	Nostoc_sp_PCC_7120	groEL	groEL1	1634	WP_010997806.1
4	Nostoc_sp_PCC_7524	groEL	groEL2	1697	WP_041555042.1
4	Nostoc_sp_PCC_7524	groES	groES1	311	WP_015139196.1
4	Nostoc_sp_PCC_7524	groEL	groEL1	1637	WP_015139197.1
4	Raphidiopsis_brookii_D9	groES	groES1	311	WP_009342971.1
4	Raphidiopsis_brookii_D9	groEL	groEL1	1637	WP_009342972.1
4	Raphidiopsis_brookii_D9	groEL	groEL2	1685	WP_009343442.1
4	Rivularia_sp_PCC_7116	groES	groES1	311	WP_015116770.1
4	Rivularia_sp_PCC_7116	groEL	groEL1	1634	WP_015116771.1
4	Rivularia_sp_PCC_7116	groEL	groEL2	1685	WP_015119171.1
4	Scytonema_PCC7110_joined	groEL	groEL2	1673	(GenBank) KYC41538.1
4	Scytonema_PCC7110_joined	groEL	groEL1	1640	(GenBank) KYC39217.1
4	Scytonema_PCC7110_joined	groES	groES1	311	(GenBank) KYC39218.1
4	Scytonema_PCC7110_joined	groES	groEL1.2	311	(GenBank) KYC38917.1
4	Scytonema_PCC7110_joined	groEL	groEL1.2	1640	(GenBank) KYC38918.1
4	Tolypothrix_sp_PCC_9009	groEL	groEL1	1637	WP_029638606.1
4	Tolypothrix_sp_PCC_9009	groES	groES1	311	WP_029638604.1
4	Tolypothrix_sp_PCC_9009	groEL	groEL2	1676	WP_029634993.1
5	Chlorogloeopsis_fritschii_PCC6912	groEL	groEL2	1661	WP_016873105.1
5	Chlorogloeopsis_fritschii_PCC6912	groES	groES1.2	329	WP_016877281.1
5	Chlorogloeopsis_fritschii_PCC6912	groEL	groEL1.2	1637	WP_016877280.1
5	Chlorogloeopsis_fritschii_PCC6912	groEL	groEL1	1640	WP_016878546.1
5	Chlorogloeopsis_fritschii_PCC6912	groES	groES1	311	WP_016878545.1
5	Chloroglopsis_PCC9212_joined	groEL	groEL2	1661	WP_016873105.1
5	Chloroglopsis_PCC9212_joined	groEL	groES1.2	1637	WP_016877280.1
5	Chloroglopsis_PCC9212_joined	groES	groEL1.2	311	WP_016877281.1
5	Chloroglopsis_PCC9212_joined	groES	groES1	311	WP_016878545.1
5	Chloroglopsis_PCC9212_joined	groEL	groEL1	1640	WP_016878546.1
5	cyanobacterium_PCC_7702	groEL	groEL2	1634	WP_017321918.1
5	cyanobacterium_PCC_7702	groES	groES1.2	311	WP_017321739.1
5	cyanobacterium_PCC_7702	groEL	groEL1.2	1637	WP_017321738.1
5	cyanobacterium_PCC_7702	groES	groES1	311	WP_017321483.1
5	cyanobacterium_PCC_7702	groEL	groEL1	1634	WP_017321482.1
5	cyanobacterium_PCC_7702	groES	not determined	311	WP_017319771.1
5	cyanobacterium_PCC_7702	groEL	not determined	1637	WP_017319770.1
5	Fischerella_sp_JSC-11	groEL	groEL2	1652	WP_009458011.1
5	Fischerella_sp_JSC-11	groES	groES1	311	WP_009457502.1
5	Fischerella_sp_JSC-11	groEL	groEL1	1637	WP_009457507.1
5	Fischerella_sp_JSC-11	groEL	groEL1.2	1634	WP_009756747.1
5	Fischerella_sp_JSC-11	groES	groES1.2	311	WP_009756748.1
5	Fischerella_sp_PCC_9339	groEL	groEL1	1637	WP_017311910.1
5	Fischerella_sp_PCC_9339	groES	groES1	311	WP_016863380.1
5	Fischerella_sp_PCC_9339	groEL	groEL2	1685	WP_017311935.1
5	Fischerella_sp_PCC_9431	groEL	groEL2	1679	WP_026722382.1
5	Fischerella_sp_PCC_9431	groES	groES1	311	WP_016863380.1
5	Fischerella_sp_PCC_9431	groEL	groEL1	1637	WP_026722402.1
5	Fischerella_sp_PCC_9605	groEL	groEL1	1637	WP_026733542.1
5	Fischerella_sp_PCC_9605	groES	groES1	335	WP_026733543.1
5	Fischerella_sp_PCC_9605	groEL	groEL2	1658	WP_026733795.1
5	Fischerella_sp_PCC_9605	groEL	groEL1.2	1631	WP_026735557.1
5	Fischerella_sp_PCC_9605	groES	groES1.2	311	WP_026735558.1
5	Fischerella_sp_PCC7521	groEL	groEL2	1652	WP_009458011.1
5	Fischerella_sp_PCC7521	groEL	groEL1	1637	WP_009457507.1
5	Fischerella_sp_PCC7521	groES	groES1	311	WP_009457502.1
5	Fischerella_sp_PCC7521	groES	groES1.2	311	WP_009756748.1
5	Fischerella_sp_PCC7521	groEL	groEL1.2	1634	WP_009756747.1
5	FisPCC73103	groEL	groEL2	1673	WP_016859319.1
5	FisPCC73103	groEL	groEL1	1637	WP_016863379.1
5	FisPCC73103	groES	groES1	311	WP_016863380.1
5	FisPCC73103	groES	groES1.2	332	WP_016863623.1
5	FisPCC73103	groEL	groEL1.2	1637	WP_016863624.1
5	FisPCC7414	groEL	groEL2	1661	WP_016865329.1
5	FisPCC7414	groEL	groEL1.2	1634	WP_016870295.1
5	FisPCC7414	groES	groES1.2	311	WP_009756748.1
5	FisPCC7414	groEL	groEL1	1637	WP_016867666.1
5	FisPCC7414	groES	groES1	311	WP_009457502.1
5	Mastigocladopsis_repens_PCC_10914	groES	groES1	335	WP_017315948.1
5	Mastigocladopsis_repens_PCC_10914	groEL	groEL1	1640	WP_017315949.1
5	Mastigocladopsis_repens_PCC_10914	groEL	groEL2	1664	WP_017316382.1

TableS2. Location of CIRCE and K-Box elements upstream of *groEL* paralogs in Stigonematales. The coordinates relate to the open reading frame beginning. Nucleotide substitutions in conserved CIRCE and K-Box elements are highlighted in red. Lacking elemnts are marked with a dash.

	Organism	Position CIRCE	Position K-Box	CIRCE	K-Box
GroESL1	Chlorogloeopsis fritschii PCC 6912	-17	-83	TTAGCACTCAGGAGTTGAGAGTGCTAA	GTTCGG TAAC CCGTACC
	Chloroglopsis sp. PCC 9212	-18	-84	TTAGCACTCAGGAGTTGAGAGTGCTAA	GTTCGG TAAC CCGTACA
	Fischerella sp. PCC 73103	-16	-82	TTAGCACTCAGGAGTTGAGAGTGCTAA	GTTCGG GAAC CCGTACT
	Fischerella sp. PCC 7414	-16	-82	TTAGCACTCAGGAGTTGAGAGTGCTAA	GTTCGG AACC CGTACG
	Fischerella sp. JSC-11	-17	-83	TTAGCACTCAGGAGTTGAGAGTGCTAA	GTTCGG GAAC CCGTACT
	Fischerella thermalis PCC 7521	-16	-82	TTAGCACTCAGGAGTTGAGAGTGCTAA	GTTCGG GAAC CCGTACC
	Fischerella sp. PCC 9339	-15	-81	TTAGCACTCAGGAGTTGAGAGTGCTAA	GTTCGG GAAC CCGTACC
	Fischerella sp. PCC 9431	-17	-83	TTAGCACTCAGGAGTTGAGAGTGCTAA	GTTCGG GAAC CCGTACG
	Fischerella sp. PCC 9605	0	-59	TTAGCACTCAGGAGTTGAGA	GTTCGG AAAC CCGTACC
	Mastigocladopsis repens PCC 10914	0	-60	TTAGCACTCAGGAGTCGAGAG	GTTCGG GAAC CCGTACA
	Cyanobacterium PCC 7702	-18	-84	TTAGCACTCAGGAACAGAGAGTGCTAA	GTTCGG GAAC CCGTACT
GroESL1.2	Chlorogloeopsis fritschii PCC 6912	-156	-221	TTAGCAATCGAAAGGTGAGAGTGCTAA	GTTCGGGAAACCNACA
	Chloroglopsis sp. PCC 9212	-44	-109	TTAGCAATCGAAAGGCGAGAGTGTTAA	GTTCGGAAAACCGCAC
	Fischerella sp. PCC 73103	-	-	-	-
	Fischerella sp. PCC 7414	-188	-296	TTAGAGTAGAACATTCAAATCTGTGGA	GTTCGGAAAACCCCAA
	Fischerella sp. JSC-11	-189	-296	TTAGAGTAGAACATTCAAATCTGTGGA	GTTCGGAAAACCCCAA
	Fischerella thermalis PCC 7521	-	-295	-	GTTCGGAAAACCCCAAA
	Fischerella sp. PCC 9605	-135	-200	TTAGCAATCGAAATAAGAGAGTGCTAA	GTTCGGCAAACCNACA
	Cyanobacterium PCC 7702	-44	-109	TTAGCAATCGAAAGGCGAGAGTGTTAA	GTTCGGAAAACCGCAC
GroEL2	Chlorogloeopsis fritschii PCC 6912	-34	-	TTAGCACTCTTTGTCTGTGAGTGCTAA	-
	Chloroglopsis sp. PCC 9212	-34	-	TTAGCACTCTTTGTCTGTGAGTGCTAA	-
	Fischerella sp. PCC 73103	-44	-	TTAGCACTCTTGCCTTGTGAGTGCTAA	-
	Fischerella sp. PCC 7414	-44	-	TTAGCACTCTTGCCTTGTGAGTGCTAA	-
	Fischerella sp. JSC-11	-44	-	TTAGCACTCTTGGCTTGTGAGTGCTAA	-
	Fischerella thermalis PCC 7521	-45	-	TTAGCACTCTTGGCTTGTGAGTGCTAA	-
	Fischerella sp. PCC 9339	-44	-	TTAGCACTCTTGCCTTGTGAGTGCTAA	-
	Fischerella sp. PCC 9431	-45	-	TTAGCACTCTTGTCTAGTGAGTGCTAA	-
	Fischerella sp. PCC 9605	-43	-	TTAGCACTCTTGTCCTGTGAGTGCTAA	-
	Mastigocladopsis repens PCC 10914	-34	-	TTAGCACTCTTGACCGCTGAGTGCTAA	-
	Cyanobacterium PCC 7702	-35	-	TTAGCACTCTTTGTCTGTGAGTGCTAA	-

TableS3. List of forward and reverse primers used for amplification and corresponding assays.

Sequence	Sequence of forward primer	Sequence of reverse primer	Assay
groES1	CTGCAGCATGGCAGCTGTATCTCTAAG	GGATCCGGGCTAACGATCGCCAAAAT	BACTH
groES1	CTGCAGCCATGGCAGCTGTATCTCTAA	GGATCCGCTAACGATCGCCAAAATGTC	BACTH
groEL1	CTGCAGCATGGCAAAGCGCATTATTTACA	GGATCCGGGTAATCGAAGTCGCCGCC	BACTH
groEL1	CTGCAGCCATGGCAAAGCGCATTATTTAC	GGATCCGTAATCGAAGTCGCCGCCG	BACTH
groES1.2	CTGCAGCCATGCTTGGAGGATTAGATATG	GGATCCTGTCAGTGTCGCAAGGATG	BACTH
groEL1.2	CTGCAGCATGGCTAAACAAATAATTTATAACG	GGATCCGGGTAATCAAAATCGCCACCCA	BACTH
groEL1.2	CTGCAGCCATGGCTAAACAAATAATTTATAACG	GGATCCGTAATCAAAATCGCCACCCA	BACTH
groEL2	CTGCAGCATGGCAAAAATTATTGCTTTGACGAAG	GGATCCGGCATCATACCCATGCCGCCC	BACTH
groEL2	CTGCAGCCATGGCAAAAATTATTGCATTTGACGAAG	GGATCCCATCATACCCATGCCGCCC	BACTH
groEL2	CTGCAGCCATGGCAAAAATTATTGCATTTGACGAAG	GGATCCCATCATACCCATGCCGCCC	BACTH
groEL1	CTGCTGGGTCTCCCATAGCTGATGGCTAATAG	GCCCGTCGGTCTCAGCGCTTTAGTAATCGAAGTCGCCGC	Complementation
groEL1.2	CTGCTGGGTCTCCTAATGTAGGAGTTAGGGGCTAG	CTGCTGGGTCTCTGCGCTTTAGTAATCAAAATCGCCAC	Complementation
groEL2	CTGGAGGGTCTCCTAATGCTGTATCCAACATCAGC	CAGCAGGGTCTCAGCGCTCTACATCATACCCATGCCGC	Complementation
groES1	GGTCTCTATGGCAGCTGTATCTCTAAGCG	GGTCTCAGCGCTTAGCTAACGATCGCCAAAATGT	Complementation
groES1.2	GGTCTCTATGCTTGGAGGATTAGATATGGCG	GGTCTCAGCGCCTATGTCAGTGTCGCAAGGATG	Complementation
pASK-IBA3sp	GCCTCACTGATTAAGCATTGGTAGG	CAGGGTTATTGTCTCATGAGCGG	Complementation
rnpB	ACCAAACTTGCTGGGTAACG	CACCTTTGCACCCTTACCTT	RT qPCR
glnA	CTGCAAGATATGCGGACAGA	AAGCCCAATTCACACTGACC	RT qPCR
groEL1	CGATTCTGCTCAAGCGTGGTA	CCAGCAGAGATGGCACCT	RT qPCR
groEL1.2	TGTTGCCCGTGCCCTTTA	ATCAAATGGTTTTTCCTTCACTCG	RT qPCR
groEL2	ATTGGCCAAATTCGCAAGCAAC	TCCGCAATTTGCGGTCTTTAAG	RT qPCR
groES1.2	AGGTGAATCAGAGGAGAAGAC	GGCGTGAAGCGTCTTCATTC	RT qPCR
pGroEL1	AGTAGAATTCGTCCTGAAGTTTCTATACAATTAC	AGTACTGCAGACAAATTTCGTGCCTTGTTAG	GFP-Fusions
pGroEL1.2	AGTAGAATTCAAAGTTCAGTTTTCTACACTCTT	AGTACTGCAGTATTTATTGAATTTACAACTCGTACTA	GFP-Fusions
pGroEL2	AGTAGAATTCTTTGCCGCTTCCATCAT	AGTACTGCAGGCCGCTTCCATCATTAC	GFP-Fusions

TableS4. Transcriptional regulation of *groEL* and *groES* paralogs in *C. fritschii* PCC 6912.

high temperature condition

Duration	Biological replicate	Ct (target gene)	Ct (endogen control)	Δct	ΔΔct	fold change
		**groEL1**	rnpB			
t(0)= calibrato	1	20,93	14,10	6,83	0,00	1,00
t(0)= calibrato	2	19,13	14,00	5,12	0,00	1,00
t(0)= calibrato	3	22,86	14,65	8,22	0,00	1,00
5 min	1	19,54	14,82	4,72	-2,11	4,31
5 min	2	16,28	13,92	2,36	-2,76	6,79
5 min	3	20,84	14,80	6,04	-2,18	4,52
15 min	1	18,57	13,69	4,87	-1,95	3,88
15 min	2	15,96	14,03	1,93	-3,20	9,18
15 min	3	19,72	15,09	4,63	-3,58	11,99
30 min	1	14,33	14,00	0,33	-6,50	90,73
30 min	2	13,43	14,17	-0,74	-5,86	58,28
30 min	3	16,57	14,75	1,81	-6,40	84,64
		**groEL1.2**	rnpB			
t(0)= calibrato	1	31,87	14,10	17,78	0,00	1,00
t(0)= calibrato	2	30,26	14,00	16,26	0,00	1,00
t(0)= calibrato	3	30,99	14,65	16,34	0,00	1,00
5 min	1	27,95	14,82	13,13	-4,65	25,05
5 min	2	25,33	13,92	11,41	-4,85	28,87
5 min	3	26,11	14,80	11,32	-5,02	32,56
15 min	1	25,39	13,69	11,70	-6,08	67,48
15 min	2	21,89	14,03	7,86	-8,40	336,68
15 min	3	24,07	15,09	8,98	-7,36	164,07
30 min	1	20,06	14,00	6,06	-11,71	3361,11
30 min	2	19,24	14,17	5,06	-11,19	2343,59
30 min	3	21,38	14,75	6,63	-9,71	836,89
		**groEL2**	rnpB			
t(0)= calibrato	1	24,15	14,10	10,05	0,00	1,00
t(0)= calibrato	2	23,97	14,00	9,97	0,00	1,00
t(0)= calibrato	3	26,32	14,65	11,67	0,00	1,00
5 min	1	21,62	14,82	6,79	-3,25	9,53
5 min	2	19,40	13,92	5,48	-4,48	22,39
5 min	3	22,36	14,80	7,57	-4,10	17,15
15 min	1	20,99	13,69	7,30	-2,75	6,73
15 min	2	20,06	14,03	6,03	-3,94	15,34
15 min	3	21,40	15,09	6,31	-5,36	41,05
30 min	1	19,17	14,00	5,17	-4,88	29,47
30 min	2	19,15	14,17	4,98	-4,99	31,76
30 min	3	20,80	14,75	6,04	-5,63	49,44

Summary

	time	0	5 min	15 min	30 min
**groEL1**	fold change	1,00	4,31	3,88	90,73
		1,00	6,79	9,18	58,28
		1,00	4,52	11,99	84,64
	mean	1,00	5,20	8,35	77,88
	SD	0,00	1,38	4,12	17,25
	time	0	5 min	15 min	30 min
**groEL1.2**	fold change	1,00	25,05	67,48	3361,11
		1,00	28,87	336,68	2343,59
		1,00	32,56	164,07	836,89
	mean	1,00	28,82	189,41	2180,53
	SD	0,00	3,75	136,38	1269,98
	time	0	5 min	15 min	30 min
**groEL2**	fold change	1,00	9,53	6,73	29,47
		1,00	22,39	15,34	31,76
		1,00	17,15	41,05	49,44
	mean	1,00	16,36	21,04	36,89
	SD	0,00	6,46	17,86	10,93

high light condition

Duration	Biological replicate	Ct (target gene)	Ct (endogen control)	Δct	ΔΔct	fold change
		**groEL1**	rnpB			
t(0)= calibrato	1	19,06	13,18	5,88	0,00	1,00
t(0)= calibrato	2	16,96	12,55	4,41	0,00	1,00
t(0)= calibrato	3	15,99	13,05	2,94	0,00	1,00
5 min	1	17,81	12,51	5,30	-0,58	1,49
5 min	2	17,40	12,93	4,47	0,05	0,96
5 min	3	16,34	12,59	3,75	0,81	0,57
15 min	1	18,02	12,93	5,09	0,68	1,73
15 min	2	17,18	13,28	3,90	-1,98	1,43
15 min	3	16,22	12,46	3,76	0,82	0,57
30 min	1	16,87	13,06	3,81	-2,07	4,21
30 min	2	17,12	12,67	4,44	0,03	0,98
30 min	3	15,93	12,93	3,00	0,06	0,96
		**groEL1.2**	rnpB			
t(0)= calibrato	1	29,46	13,18	16,28	0,00	1,00
t(0)= calibrato	2	30,27	12,55	17,73	0,00	1,00
t(0)= calibrato	3	28,83	13,05	15,77	0,00	1,00
5 min	1	28,25	12,51	15,74	-0,54	1,45
5 min	2	28,97	12,93	16,04	-1,69	3,22
5 min	3	27,11	12,59	14,52	-1,25	2,38
15 min	1	28,01	12,93	15,09	-2,64	2,29
15 min	2	27,70	13,28	14,42	-1,86	9,92
15 min	3	27,69	12,46	15,23	-0,55	1,46
30 min	1	26,27	13,06	13,21	-3,07	8,39
30 min	2	27,32	12,67	14,65	-3,08	8,47
30 min	3	26,25	12,93	13,33	-2,45	5,45
		**groEL2**	rnpB			
t(0)= calibrato	1	23,79	13,18	10,61	0,00	1,00
t(0)= calibrato	2	23,41	12,55	10,86	0,00	1,00
t(0)= calibrato	3	22,49	13,05	9,44	0,00	1,00
5 min	1	20,17	12,51	7,67	-2,94	7,68
5 min	2	20,52	12,93	7,58	-3,28	9,71
5 min	3	19,13	12,59	6,54	-2,90	7,46
15 min	1	20,57	12,93	7,65	-2,96	7,79
15 min	2	19,84	13,28	6,56	-4,30	19,69
15 min	3	19,79	12,46	7,33	-2,11	4,32
30 min	1	19,54	13,06	6,48	-4,13	17,49
30 min	2	20,23	12,67	7,56	-3,30	9,83
30 min	3	19,28	12,93	6,35	-3,08	8,48

Summary

	time	0	5 min	15 min	30 min
**groEL1**	fold change	1,00	1,49	1,73	4,21
		1,00	0,96	1,43	0,98
		1,00	0,57	0,57	0,96
	mean	1,00	1,01	1,24	2,05
	SD	0,00	0,46	0,60	1,87
	time	0	5 min	15 min	30 min
**groEL1.2**	fold change	1,00	1,45	2,29	8,39
		1,00	3,22	9,92	8,47
		1,00	2,38	1,46	5,45
	mean	1,00	2,35	4,55	7,43
	SD	0,00	0,88	4,66	1,72
	time	0	5 min	15 min	30 min
**groEL2**	fold change	1,00	7,68	7,79	17,49
		1,00	9,71	19,69	9,83
		1,00	7,46	4,32	8,48
	mean	1,00	8,28	10,60	11,94
	SD	0,00	1,24	8,06	4,86

diazotrophic condition (nitrogen deprivation)

Duration	Biological replicate	Ct (target gene)	Ct (endogen control)	Δct	ΔΔct	fold change
		**groEL1**	rnpB			
t(0)= calibrato	1	20,06	14,00	6,05	0,00	1,00
t(0)= calibrato	2	21,95	14,90	7,06	0,00	1,00
t(0)= calibrato	3	20,03	15,17	4,86	0,00	1,00
3 h	1	17,02	15,90	1,12	-4,93	30,56
3 h	2	23,43	20,58	2,85	-4,20	18,40
3 h	3	17,77	17,77	0,00	-4,86	28,96
8 h	1	19,12	15,87	3,24	-2,81	7,02
8 h	2	20,56	16,68	3,88	-3,17	9,02
8 h	3	21,53	17,28	4,25	-0,61	1,52
24 h	1	22,11	19,23	2,88	-3,17	9,02
24 h	2	20,73	15,94	4,79	-2,27	4,82
24 h	3	19,93	18,09	1,84	-3,02	8,11
		**groEL1.2**	rnpB			
t(0)= calibrato	1	29,10	14,00	15,09	0,00	1,00
t(0)= calibrato	2	29,93	14,90	15,03	0,00	1,00
t(0)= calibrato	3	29,46	15,17	14,29	0,00	1,00
3 h	1	27,42	15,90	11,52	-3,58	11,92
3 h	2	31,37	20,58	10,79	-4,24	18,93
3 h	3	27,60	17,77	9,82	-4,46	22,05
8 h	1	25,87	15,87	9,99	-5,10	34,33
8 h	2	24,98	15,27	9,70	-5,33	40,13
8 h	3	27,16	17,28	9,88	-4,40	21,15
24 h	1	28,96	19,23	9,73	-5,37	41,25
24 h	2	26,92	18,09	8,84	-6,19	73,04
24 h	3	27,59	18,09	9,50	-4,79	27,66
		**groEL2**	rnpB			
t(0)= calibrato	1	24,76	14,00	10,75	0,00	1,00
t(0)= calibrato	2	25,88	14,90	10,99	0,00	1,00
t(0)= calibrato	3	24,47	15,17	9,29	0,00	1,00
3 h	1	23,97	15,90	8,07	-2,68	6,43
3 h	2	28,76	20,58	8,18	-2,80	6,98
3 h	3	24,30	17,77	6,53	-2,76	6,78
8 h	1	25,35	15,87	9,48	-1,28	2,42
8 h	2	25,27	15,27	9,99	-0,99	1,99
8 h	3	28,14	17,28	10,86	1,57	0,34
24 h	1	27,90	19,23	8,67	-2,08	4,23
24 h	2	27,31	18,09	9,23	-1,76	3,39
24 h	3	27,18	18,09	9,08	-0,21	1,16
		**glnA**	rnpB			
t(0)= calibrato	1	19,10	15,19	3,91	0,00	1,00
t(0)= calibrato	2	20,99	12,77	8,21	0,00	1,00
t(0)= calibrato	3	22,78	16,40	6,39	0,00	1,00
3 h	1	15,65	15,20	0,45	-3,46	11,02
3 h	2	24,16	19,46	4,70	-3,52	11,43
3 h	3	16,22	12,73	3,49	-2,90	7,46
8 h	1	18,29	13,66	4,63	0,72	0,61
8 h	2	20,03	13,23	6,80	-1,42	2,67
8 h	3	19,92	11,95	7,97	1,59	0,33
24 h	1	19,48	14,38	5,10	1,19	0,44
24 h	2	19,65	12,79	6,86	-1,35	2,55
24 h	3	18,56	12,56	6,00	-0,39	1,31

Summary

	time	0	3 h	8 h	24 h
**groEL1**	fold change	1,00	30,56	7,02	9,02
		1,00	18,40	9,02	4,82
		1,00	28,96	1,52	8,11
	mean	1,00	25,97	5,86	7,32
	SD	0,00	6,61	3,88	2,21
**groEL1.2**	time	0	3 h	8 h	24 h
	fold change	1,00	11,92	34,33	41,25
		1,00	18,93	40,13	73,04
		1,00	22,05	21,15	27,66
	mean	1,00	17,63	31,87	47,32
	SD	0,00	5,19	9,73	23,29
**groEL2**	time	0	3 h	8 h	24 h
	fold change	1,00	6,43	2,42	4,23
		1,00	6,98	1,99	3,39
		1,00	6,78	0,34	1,16
	mean	1,00	6,73	1,58	2,93
	SD	0,00	0,28	1,10	1,59
**glnA**	time	0	3 h	8 h	24 h
	fold change	1,00	11,02	0,61	0,44
		1,00	11,43	2,67	2,55
		1,00	7,46	0,33	1,31
	mean	1,00	9,97	1,21	1,43
	SD	0,00	2,19	1,28	1,06

urve parameters (y=mx+b)

	groEL1	groEL1.2	groEL2
amplicon leng	121	100	151
MW(amplicon	37361,25	30991,09	46924,53
m = slope	-3,39	-3,39	-3,45
b = intercept(y	6,99	7,10	8,10
R^2	1,00	1,00	1,00
primer efficier	1,97	1,97	1,95

MW=Molecular weight

R^2=coefficient of determination

x=ng cDNA

	Biological replicate	Ct	log	pg cDNA	molecules/ µl	copies in 20µl	copy number/ ng RNA
**groEL1**	1	20,06	-3,85	1,40E-04	4,21E+10	8,41E+11	1,68E+10
	2	18,22	-3,31	4,89E-04	7,88E+06	1,58E+08	3,15E+06
	3	20,22	-3,90	1,26E-04	2,03E+06	4,05E+07	8,10E+05
	4	19,06	-3,56	2,76E-04	4,45E+06	8,90E+07	1,78E+06
	5	20,93	-4,11	7,78E-05	1,25E+06	2,51E+07	5,01E+05
	6	19,13	-3,58	2,64E-04	4,26E+06	8,52E+07	1,70E+06
**groEL1.2**	1	29,46	-6,63	2,37E-07	3,82E+03	7,64E+04	2,00E+03
	2	28,83	-6,44	3,64E-07	5,87E+03	1,17E+05	3,07E+03
	3	29,46	-6,63	2,37E-07	3,82E+03	7,63E+04	2,00E+03
	4	29,10	-6,52	3,03E-07	4,88E+03	9,77E+04	2,55E+03
	5	29,93	-6,76	1,73E-07	2,78E+03	5,57E+04	1,46E+03
	6	29,42	-6,61	2,43E-07	3,91E+03	7,83E+04	2,05E+03
**groEL2**	1	24,15	-5,06	8,75E-06	1,41E+05	2,82E+06	1,14E+05
	2	23,41	-4,84	1,44E-05	2,33E+05	4,66E+06	1,87E+05
	3	24,47	-5,15	7,03E-06	1,13E+05	2,27E+06	9,19E+04
	4	23,79	-4,95	1,12E-05	1,80E+05	3,60E+06	1,45E+05
	5	24,76	-5,24	5,78E-06	9,32E+04	1,86E+06	7,59E+04
	6	23,97	-5,01	9,86E-06	1,59E+05	3,18E+06	1,28E+05

Transcription level groESL

Biological replicate	Ct (target gene)	Ct (endogen control)	Δct	Ratio groEL:groES
	groEL1.2	rnpB		
1	27,50953851	15,4515928	12,0579457	
2	28,1796575	15,9387998	12,2408577	
3	27,6302689	15,4038369	12,226432	

	groES1.2	rnpB		
1	24,5800028	15,4515928	9,12841003	1,32092508
2	25,6518929	15,9387998	9,71309315	1,26024301
3	25,5089216	15,4038369	10,1050847	1,2099287
